# 7-*O*-Carboxylic Acid-Substituted 3-*O*-Alkyl Difluoroquercetin; An Aztreonam-Potentiating Agent Against Carbapenemase-Producing *Pseudomonas aeruginosa* Through Simultaneous Inhibition of Metallo-β-Lactamase and Efflux Pump

**DOI:** 10.3390/antibiotics13121202

**Published:** 2024-12-10

**Authors:** Seongyeon Lee, Taegum Lee, Mi Kyoung Kim, Joong Hoon Ahn, Seri Jeong, Ki-Ho Park, Youhoon Chong

**Affiliations:** 1 Department of Bioscience and Biotechnology, Bio/Molecular Informatics Center, Konkuk University, Hwayang-dong, Gwangjin-gu, Seoul 143-701, Republic of Korea; tjddus98@konkuk.ac.kr (S.L.); co4121@konkuk.ac.kr (T.L.); mkkim@konkuk.ac.kr (M.K.K.); jhahn@konkuk.ac.kr (J.H.A.); 2 Department of Laboratory Medicine, Hallym University College of Medicine, Chuncheon 24252, Republic of Korea; hehebox@naver.com; 3 Department of Infectious Disease, Kyung Hee University School of Medicine, Seoul 02447, Republic of Korea

**Keywords:** *Pseudomonas aeruginosa*, metallo-β-lactamase, efflux pump, simultaneous inhibition, aztreonam, potentiation

## Abstract

**Background/Objectives:** Previously, we reported that 3-*O*-alkyl difluoroquercetins (di-F-Q) potentiates the antimicrobial activity of aztreonam (ATM) against metallo-β-lactamase (MBL)-producing *P. aeruginosa* through simultaneous inhibition of MBLs and efflux pumps. However, the ATM-potentiating activity of the 3-*O*-alkyl di-F-Q was observed only at high and potentially toxic concentrations (32 mg/L). **Methods:** As both MBLs and efflux pumps reside in the periplasm of Gram-negative bacteria, their inhibitors should accumulate in the periplasmic space. However, the outer membrane porins, the major entry pathway in Gram-negative bacteria, allow the passive diffusion of hydrophilic polar molecules across the outer membrane. Thus, we reasoned that the introduction of a polar substituent at 7-OH position of 3-*O*-alkyl di-F-Q would enhance its periplasmic concentration to result in potentiation of ATM at lower concentrations. **Results:** The title compound **5** exhibited inhibitory activity against NDM-1 as well as the efflux pump of *P. aeruginosa*, which resulted in synergistical potentiation of ATM. A combination of ATM (8 mg/L) and **5** (8 mg/L) inhibited 80% of the ATM-resistant CPPA, while ATM alone did not show any inhibition. In addition, only 4 mg/L of **5** was needed to reduce the MIC_90_ of ATM four-fold in ATM-resistant CPPA (n = 15). The time–kill data further supported the effectiveness of the combined treatment of ATM with **5**, and the combination of ATM (1xMIC) with 8 mg/L of **5** showed bactericidal effects in every bacterial strain tested (PA-002, *bla*_IMP_, PA-003, *bla*_VIM_, PA-014, *bla*_GES_, and PA-017, *bla*_NDM_) by reducing the bacterial loads by 5.1 log_10_~8.9 log_10_. **Conclusions:** The title compound **5** exhibited inhibitory activity against NDM-1 as well as the efflux pump of *P. aeruginosa*, and the combined inhibitory activity resulted in synergistical potentiation of ATM. It should be noted that most CPPA isolates tested were sensitized to 8 mg/L of ATM upon combination with 4~8 mg/L of **5**.

## 1. Introduction

β-Lactams (BLs), a class of antibiotics with a highly reactive 4-membered cyclic amide ring, are classified into penicillins, cephalosporins, monobactams, and carbapenems, and they are the most commonly prescribed antibiotics [1]. However, like other antibiotics, BLs suffer from multidrug resistance (MDR) [2] and β-lactam-hydrolyzing enzymes (β-lactamases) are known to be a key factor of resistance [3,4]. In the context of MDR, carbapenems are of particular interest because of their broadest spectrum of activity as well as less vulnerability to most BL resistance determinants, and thus, they are often considered as a last-resort antibiotic for severe and complicated MDR infections [5]. Unfortunately, resistance to carbapenems has emerged, and the World Health Organization has announced carbapenem-resistant Gram-negative bacteria such as *Acinetobacter baumannii*, Enterobacteriaceae (CRE), and *Pseudomonas aeruginosa* as a critical or high-risk group on their bacterial priority pathogens list in 2024 [6]. The antimicrobials effective against these carbapenem-resistant Gram-negative MDR infections are limited, which has led to the use of previously abandoned toxic antibiotics such as polymyxins and aminoglycosides [7,8]. Therefore, there is a clear need to develop potent and less toxic antimicrobials to treat patients with carbapenem-resistant MDR infections.

Carbapenem resistance has been attributed to many resistance mechanisms, but the production of carbapenem-hydrolyzing β-lactamases (carbapenemases) is known to be most critical. Development of β-lactamase inhibitors (BLis) has, thus, been attempted, and the use of BLis to potentiate BLs against Gram-negative organisms has been clinically validated by the recently approved BL/BLi combinations such as FEP/EMT [9], SUL/DUR [10], IMP/CLN/REL [11], MEM/VAB [12], and CAZ/AVI [13] (Table S1, Supporting Information, [14,15]). However, BLis approved so far have inhibitory activity against class A/C enzymes but they are not able to inhibit the class B metallo-β-lactamase (MBLs) which are characterized by the Zn^2+^ ions at the active center. One of the most significant concerns about MBLs is their carbapenemases activities [16,17], and they are found in about 22% of carbapenem non-susceptible strains worldwide [18]. Over time, the frequency and distribution of the MBLs have continuously increased, and they are often found in the Gram-negative bacteria such as Enterobacterales, *P. aeruginosa*, and *A. baumannii*. Several broad-spectrum BLis, such as taniborbactam [19], zidebactam [20], and xeruborbactam [21], also showed MBL inhibitory activity and they are under clinical development (Table S1). Nevertheless, the treatment of infections caused by carbapenem-resistant MBL-producing Gram-negative bacteria remains as a significant unmet medical need.

On the other hand, the monobactam antibiotics aztreonam (ATM) provides alternative option in overcoming the MBL-induced carbapenemases resistance because ATM does not serve as a substrate for class B MBLs [22]. However, MBL-producing Gram-negative strains co-produce multiple β-lactamases such as extended spectrum β-lactamase (ESBL) and AmpC β-lactamase, which readily hydrolyze ATM [23]. As these ATM-hydrolyzing class A/C/D β-lactamases can be inhibited by avibactam (AVI), a combination of ATM and AVI was proposed, which was shown to be highly effective in treating infections caused by MBL-producing Enterobacteriaceae [24,25]. However, unlike the *Enterobacterales*, most MBL-positive *P. aeruginosa* strains remain resistant to ATM/AVI combination [26,27,28], which might be attributed to hyperactive efflux systems, production of variants of *Pseudomonas*-derived cephalosporinases (PDC), or impermeability conferred by mutation in *oprD* [29,30,31]. Among those, mutations affecting negative regulators of the *mexAB-oprM* efflux system (*mexR* and *nalD*) were the most frequently observed factor affecting ATM resistance in *P. aeruginosa* [32,33,34,35].

Based on these observations, we have previously proposed that simultaneous inhibition of β-lactamases and efflux pumps would revive ATM against MBL-producing *P. aeruginosa*, which led to the identification of the 3-*O*-alkyl difluoroquercetin (3-*O*-alkyl di-F-Q) derivatives as novel multitarget inhibitors of broad-spectrum β-lactamases including MBLs and efflux pumps [36]. As anticipated, the multitarget inhibitor potentiated the antimicrobial activity of ATM, and more than four-fold reduction in MIC of ATM was observed in 19 out of 21 MBL-producing *P. aeruginosa* clinical strains [36]. However, the ATM-potentiating activity of the 3-*O*-alkyl di-F-Q derivatives was observed only at high and potentially toxic concentrations (32 mg/L). Therefore, in this study, we attempted to enhance the ATM-potentiating activity of the 3-*O*-alkyl di-F-Q derivatives through structural modification. We have been particularly interested in the high hydrophobicity of the 3-*O*-alkyl di-F-Q derivatives, because most antibiotics active against the Gram-negative bacteria have a highly polar nature [37,38,39,40]. Thus, through the introduction of a polar substituent at the 7-OH position, we prepared a series of 3,7-bis-*O*-substituted di-F-Q derivatives and investigated their ATM-potentiating activity against the MBL-producing carbapenem-resistant clinical *P. aeruginosa* strains under in vitro as well as in vivo settings.

## 2. Results

### 2.1. Design and Synthesis of the Title Compounds, 3,7-Bis-O-Substituted Di-F-Q Derivatives

#### 2.1.1. Design

Both MBL-catalyzed hydrolysis reactions and efflux-mediated antimicrobial resistance take place in the periplasm of Gram-negative bacteria. Thus, the simultaneous inhibitors of MBL and efflux pumps should penetrate into the periplasmic space through the outer membrane [37,38]. However, in Gram-negative bacteria, the outer membrane protein porins are major entry pathway which form transmembrane pores to allow the passive diffusion of hydrophilic polar molecule across the outer membrane. In addition, regulation of porin-mediated outer membrane permeability works in coordination with efflux, and active drug efflux is considered as the main cause of intrinsic and acquired drug resistance of Gram-negative bacteria to result in low periplasmic accumulation of antibiotics as well as BLis [37,38,39,40]. These observations collectively suggest that focusing on the passage through porins is the crucial step in designing a drug against Gram-negative bacteria, and studies have confirmed that drugs with appropriate physicochemical properties tend to accumulate more in Gram-negatives: zwitterionic molecules have increased penetration through porins while dianionic and/or bulky compounds with protruding substituents have reduced penetration [41]. As such, antimicrobials acting against Gram-negative bacteria have remarkably different physicochemical properties compared with drugs of other therapeutic areas [41]. Molecular weight (MW) and lipophilicity are two major properties of the antibacterials active against Gram-negative organisms, and they have large MW cutoff (~600 Da) and high polarity [mean clogD_7.4_ = −2.8, mean polar surface area (PSA) = 165 Å^2^] [42]. In this context, the previously identified multitarget inhibitors, 3-*O*-alkyl di-F-Q derivatives [36], have suboptimal values of clogD_7.4_ (2.56~3.13) and PSA (76.0 Å^2^), which indicates that introduction of polar substituents into this scaffold would potentiate their activity to potentiate antibiotics against Gram-negative bacteria.

#### 2.1.2. Synthesis

**Scheme 1:**
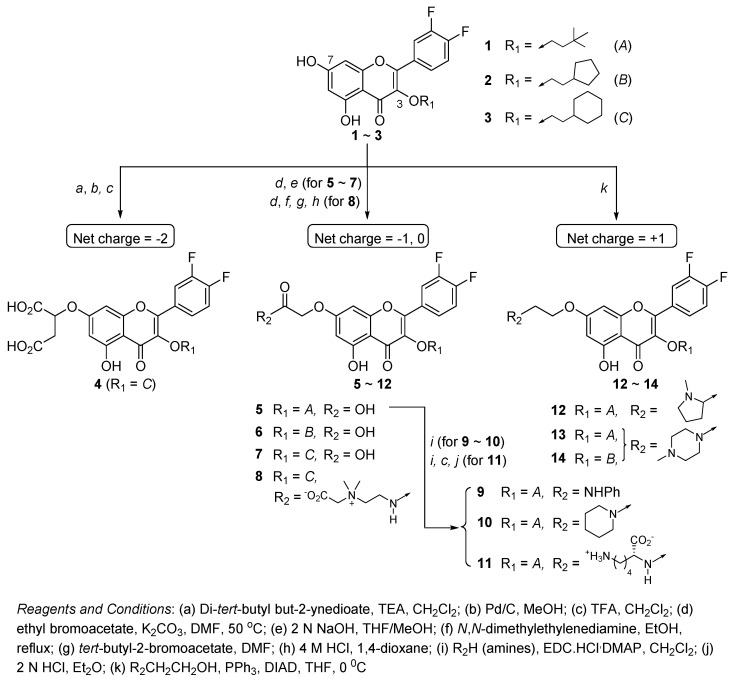
Syntheses of the title compounds, 3,7-bis-*O*-substituted di-F-Q derivatives (**4**~**14**), starting from 3-*O*-substituted di-F-Q (**1**~**3**).

The previously reported 3-*O*-alkyl di-F-Q derivatives (**1**~**3**, Scheme 1) with ATM-potentiating activity [36] commonly have a free 7-OH group, and a polar substituent at this position could be readily introduced (Scheme 1). In order to investigate the influence of charges on the activity, substituents with various charges were tested. Thus, dicarboxylic acid (**4**, charge = −2), α-aryloxy acetates (**5**~**7**, charge = −1), α-aryloxy acetamides (**8**~**11**, charge = 0), and ethanolamines (**12**~**14**, charge = +1) derivatives were prepared by nucleophilic addition, nucleophilic substitution, acyl substitution, or Mitsunobu reaction of the 3-*O*-alkyl di-F-Q derivatives (**1**~**3**) (Scheme 1). Briefly, nucleophilic addition of **3** to di-*tert*-butyl but-2-ynedioate provided the corresponding 7-*O*-enediester, which was converted to **4** after reduction followed by acidic hydrolysis. Nucleophilic substitution of **1**~**3** with ethyl bromoacetate was achieved to generate the corresponding α-aryloxy acetic acid ethyl esters, which were either hydrolyzed to the acetic acid salts (**5**~**7**) or transformed into the α-aryloxy acetamide (**8**) after direct amidation with *N*,*N*-dimethylethylenediamine followed by further substitution with 2-bromoacetate. Other α-aryloxy acetamide (**9**~**11**) were prepared by EDC [1-ethyl-3-(3-dimethylaminopropyl)carbodiimide]-coupling of **5** with various amines. Among the series, two α-aryloxy acetamides (**8** and **11**) have zwitterionic characters. On the other hand, the Mitsunobu coupling reaction of **1** or **2** with *N*-substituted ethanolamines produced the positively charged derivatives **12**~**14**.

### 2.2. Multitarget Inhibition Activity of 3,7-Bis-O-Substituted Di-F-Q Derivatives

#### 2.2.1. Inhibitory Activity Against Various β-Lactamases

In this study, we attempted to modulate physicochemical properties of the previously reported multitarget inhibitors (**1**~**3**) through introduction of a substituent at 7-*O* position. The newly synthesized 3,7-bis-*O*-substituted di-F-Q derivatives (**4**~**14**) possess a wide range of polarity properties, which are represented by net charges (−2~+1), cLogD_7.4_ (−1.63~5.69), and PSA (161.9~68.2 Å^2^) (Table 1).

Previously, we have identified the di-F-Q derivatives with bulky hydrophobic groups tethered at the 3-*O* position via an ethylene linker (**1**~**3**, Scheme 1) as pan-β-lactamase inhibitors with IC_50_ values of 15.3~21.0, 11.3~17.3, 6.3~14.8, 4.4~11.9, and 10.1~15.9 μM for AmpC, KPC-2, OXA-48, VIM-2, and NDM-1, respectively (Table 1) [36]. However, the introduction of substituents at the 7-*O* position resulted in abrogation of the inhibitory activity against AmpC (Table 1). Except the 7-*O*-acetamido derivatives **9**, other derivatives were also inactive against KPC-2 as well as OXA-48 (Table 1). However, the α-aryloxy acetates (**5**~**7**) showed preferential inhibitory activity against the MBLs (VIM-2 and NDM-1) (Table 1). Among the series, only **9** showed broad-spectrum inhibitory activity against KPC-2, OXA-48, and MBLs (Table 1). The structure–activity relationships observed in Table 1 suggests that the binding sites of the 7-*O* substituents of the 3,7-bis-*O*-substituted di-F-Q derivatives in the MBLs are short and narrow, which are big enough to accommodate acetates (**5**~**7**) and acetanilide (**9**). Thus, the 3,7-bis-*O*-substituted di-F-Q derivatives with a long (**8**, **11**) or bulky (**10**) 7-*O* substituent fail to bind to the MBLs. The broad-spectrum β-lactamase inhibitory activity of **9** also suggests that the β-lactamases share the same binding site to the 7-*O*-acetanilide functionality.

#### 2.2.2. Inhibitory Activity Against Efflux Pumps

**Figure 1:**
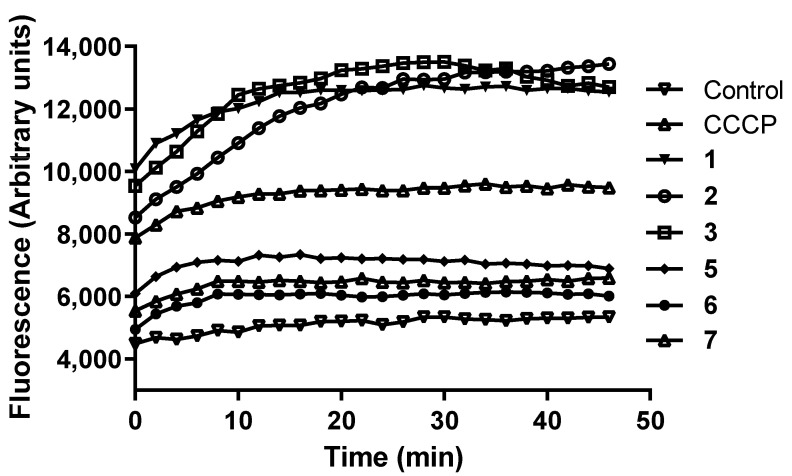
EtBr fluorescence from PA-017, *bla*_NDM_ in the absence of efflux pump inhibitors (control) and in the presence of 8 mg/L of CCCP, 3-*O*-alkyl di-F-Q derivatives (**1**~**3**) and 3,7-bis-*O*-substituted di-F-Q derivatives (**5**~**7**).

The title compounds were also evaluated for their ability to inhibit efflux pump activity in a clinical strain of NDM-1-producing *P. aeruginosa* (PA-017, *bla*_NDM_; Table 1). While the previously reported efflux pump inhibitors **1**~**3** remarkably enhanced the fluorescence emitting from EtBr (ethidium bromide) accumulated inside the bacterial cell (Table 1, Figure 1), the introduction of a substituent at 7-*O* position resulted in significant loss of the EtBr fluorescence (Table 1). The α-aryloxy acetates (**5**~**7**) were the only exceptions, but their EtBr fluorescence-enhancing activity was only marginal, which were weaker than that of the positive control, CCCP (carbonyl cyanide 3-chlorophenylhydrazone) (Figure 1). Unlike the α-aryloxy acetates (**5**~**7**), acetanilide (**9**) did not show efflux pump inhibitory activity, and this result indicates that the efflux pumps have very limited size that can discriminate acetates (**5**~**7**) and acetanilide (**9**).

### 2.3. Potentiation of Antimicrobial Activity of ATM

#### 2.3.1. ATM-Potentiating Activity of the Synthesized Compounds in NDM-1-Producing *P. aeruginosa* (PA-017, bla_NDM_)

In general, the 7-*O*-substitutions on the 3-*O*-alkyl di-F-Q scaffold were accompanied by loss of inhibitory activity against β-lactamases (Table 1). However, the α-aryloxy acetates (**5**~**7**) and α-aryloxy acetamide (**9**~**10**) showed MBL-specific inhibitory activity, and their IC_50_ values against NDM-1 (6.1~16.1 μM) were comparable to those of the 3-*O*-alkyl di-F-Q derivatives (**1**~**3**) (10.1~15.9 μM). Thus, we examined the antibiotic-potentiating activity of the title compounds (**4**~**14**) in NDM-producing *P. aeruginosa*. Interestingly, upon combination with ATM, the α-aryloxy acetates (**5**~**7**) potentiated the antimicrobial activity of ATM more efficiently than **1** (Table 1); in a clinical strain of NDM-1-producing *P. aeruginosa* (PA-017, *bla*_NDM_), the derivatives **5**~**7** reduced MIC of ATM four- to eight-fold at concentrations (16 mg/L) even lower than those required by **3** (32 mg/L) to potentiate ATM four-fold (Table 1). In contrast, the α-aryloxy acetamides (**9**~**10**) with similar IC_50_s against NDM-1 (7.1~7.6 μM) did not show any ATM-potentiating activity (Table 1). This difference in ATM-potentiating activity between the α-aryloxy acetates (**5**~**7**) and α-aryloxy acetamide (**9**~**10**) might be attributed to the simultaneous inhibition of the efflux pumps: in the series of the compounds synthesized, only **5**~**7** were found to inhibit, albeit weakly, efflux pump activity (Table 1 and Figure 1).

#### 2.3.2. ATM-Potentiating Activity of 5 in Clinical Isolates of Carbapenemase-Producing *P. aeruginosa* (CPPA)

Among the 14 di-F-Q derivatives, compound **5** demonstrated the most effective aztreonam (ATM)-potentiating activity against NDM-1-producing *P. aeruginosa* (PA-017), resulting in an eight-fold decrease in the MIC of ATM when combined with compound **5**. This may be attributed to its highly effective inhibitory effect on NDM-1, with an IC_50_ of 6.1 ± 0.4 μM (Table 1). The ATM-potentiating activity of **5** was further investigated in 26 clinical isolates of *P. aeruginosa* producing various carbapenemases, which were composed of three GES-producers (class A carbapenemase), one VIM-producer (class B MBL), seven IMP-producers (class B MBL), thirteen NDM-producers (class B MBL), and two co-producers of IMP and NDM (Table S2, Supporting Information). Antimicrobial susceptibility of these carbapenemases-producing *P. aeruginosa* (CPPA) strains showed high resistance to most antibiotics tested (73~96%; Table S2, Supporting Information). However, the 26 clinical CPPA isolates showed different susceptibility to ATM; 7 showed resistance to ATM (MIC ≥ 32 mg/L), while 8 and 11 strains were categorized as intermediate (MIC = 16 mg/L) and sensitive (MIC ≤ 8 mg/L), respectively. Therefore, these strains are appropriately organized to monitor the ATM-potentiating effect of the title compound **5** in *P. aeruginosa* with different levels of resistance.

Upon combination with **5**, potentiation of ATM was observed, which resulted in a significant reduction of MICs of ATM (Table 2). Combination of ATM with **5** resulted in sensitization of the most clinical strains of *P. aeruginosa* to ATM (MIC ≤ 8 mg/L in 25 out of 26), and 14 out of 15 ATM-non-susceptible pathogens (MIC ≥ 16 mg/L) were converted into sensitive ones (Table 2). In addition, among 11 ATM-susceptible strains, 8 showed more than four-fold sensitization to ATM (four-fold reduction in MICs of ATM). Taken together, in 22 out of 26 CPPA isolates (85%), ATM/**5** combination showed synergistic effects. Noteworthy is the fact that, in most cases, 4~16 mg/L of **5** was enough to show synergy with ATM.

**Figure 2:**
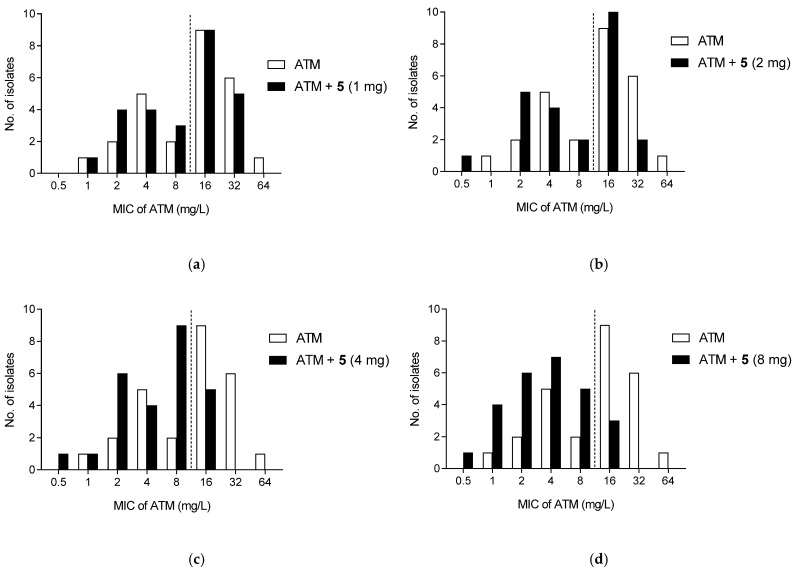

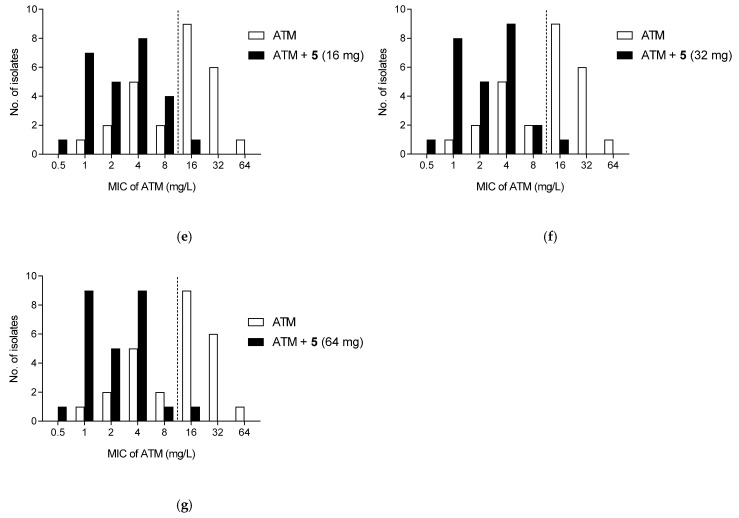
MIC distributions of ATM alone and ATM/**5** combinations with different concentrations of **5**: (**a**) 1 mg/L, (**b**) 2 mg/L, (**c**) 4 mg/L, (**d**) 8 mg/L, (**e**) 16 mg/L, (**f**) 32 mg/L, and (**g**) 64 mg/L. The dashed lines indicate clinical breakpoints of ATM (8 mg/L).

Compared with the MICs of ATM used alone, the MIC distributions of ATM in combination with **5** are left-shifted, and the shift became more distinct as the concentration of **5** increased (Figure 2). Further analysis of the data showed that, in all tested CPPA isolates (n = 26), the percentage of inhibition by ATM at its clinical breakpoint (8 mg/L) increased from 38% to 88% by addition of 8 mg/L of **5** (Table S3, Supporting Information). In ATM-resistant CPPA isolates (n = 15), a dramatic change in the percentage of inhibition by ATM was observed as ATM (8 mg/L) was combined with **5**; ATM/**5** (8 mg/L + 8 mg/L) inhibited 80% of the ATM-resistant CPPA, while ATM alone did not show any inhibition (Table S3, Supporting Information).

The cumulative percentage of inhibition of the CPPA isolates also shows that, when combined with 4 mg/L and 8 mg/L of **5**, ATM at its clinical breakpoint (8 mg/L) is enough to inhibit more than 80% of all the CPPA isolates (n = 26) and 15 ATM-resistant CPPA isolates, respectively (Figures S1 and S2 and Table S3, Supporting Information).

The MIC_50_ and MIC_90_ of ATM were also determined to show that these values remarkably decrease in accordance with increasing concentrations of **5** (Table 3). Thus, in all tested CPPA isolates (n = 26), four-fold reductions in MIC_50_ and MIC_90_ were observed upon addition of 8 mg/L and 16 mg/L of **5**, respectively. Of note, only 4 mg/L of **5** was needed to reduce the MIC_90_ of ATM four-fold in ATM-resistant CPPA (n = 15).

Table 4 compares the antibacterial efficacy of ATM-**5** with other β-lactam/β-lactamase inhibitors against 26 CPPA isolates. The MIC_50_ and MIC_90_ of ATM combined with 8 mg/L of **5** were 4 and 16 mg/L, respectively, which are lower than those of ceftazidime-avibactam (16 and 64 mg/L), ceftolozane-tazobactam (32 and 128 mg/L), and imipenem-relebactam (64 and 256 mg/L). The susceptibility of the tested pathogens to ATM combined with 8 mg/L of **5** was 88%, which was significantly higher than those to ceftazidime-avibactam (14%), ceftolozane-tazobactam (10%), and imipenem-relebactam (14%).

#### 2.3.3. Time–Kill Studies

**Figure 3:**
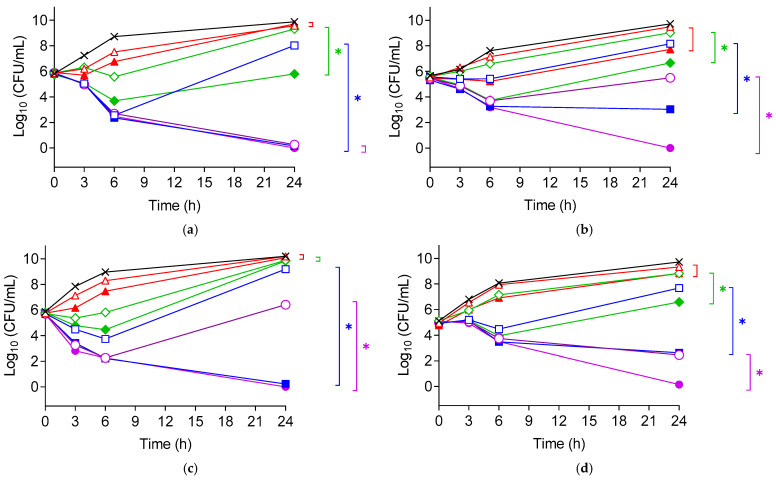

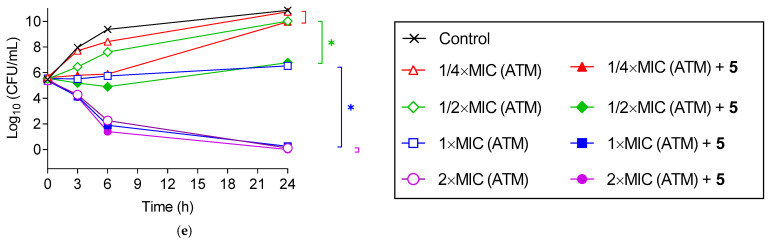
Synergistic time–kill curves for ATM with and without addition of **5** against (**a**) wild-type *P. aeruginosa* (ATCC 27853) and CPPA isolates producing (**b**) IMP (PA-002, *bla*_IMP_), (**c**) VIM (PA-003, *bla*_VIM_), (**d**) GES (PA-014, *bla*_GES_), and (**e**) NDM (PA-017, *bla*_NDM_). Asterisks indicates the synergistic killing activity. The error bars were omitted for clarity because they were shorter than the size of the symbols.

Time–kill assays were performed by exposing wild-type *P. aeruginosa* (ATCC 27853) and CPPA isolates (PA-002, *bla*_IMP_, PA-003, *bla*_VIM_, PA-014, *bla*_GES_, and PA-017, *bla*_NDM_) to two-fold concentrations from 1/4 to 2 times the MIC of ATM in the absence and presence of 8 mg/L of **5** for 24 h. After combination with **5**, significant reductions in the mean bacterial loads (log_10_ CFU/mL) were observed for wild-type *P. aeruginosa* (ATCC 27853) and CPPA isolates treated with ATM (Figure 3). Of note, at concentrations higher than 1 MIC, ATM exhibited bactericidal activity in all *P. aeruginosa* strains tested upon combination with **5** (8 mg/L) (Figure 3).

The time–kill data were further analyzed to show the effectiveness of ATM in combination with **5**. At 1xMIC, ATM failed to reduce the bacterial loads in all *P. aeruginosa* strains including the wild-type ATCC 27853 (Table 5). However, upon combination with 8 mg/L of **5**, ATM (1xMIC) showed bactericidal effects in every bacterial strain tested, and the combination of ATM (1xMIC) and **5** (8 mg/L) reduced the bacterial loads by 5.1 log_10_~8.9 log_10_ (Table 5).

#### 2.3.4. Effects of the Addition of Compound **5** to Subinhibitory ATM on Bacterial Morphology

**Figure 4:**
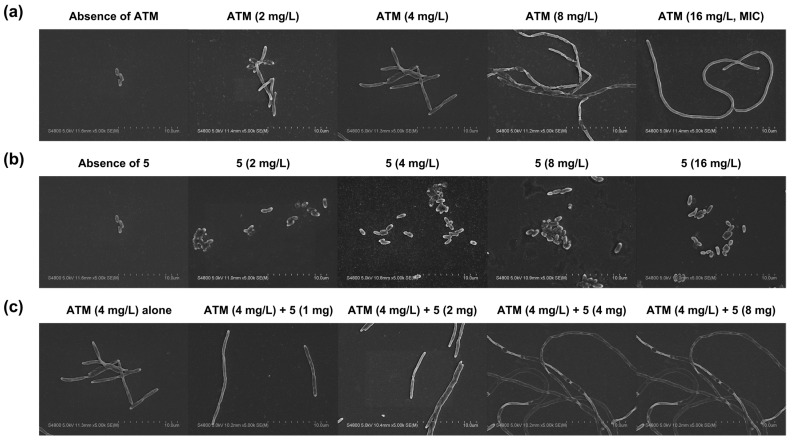
Scanning electron microscopy micrographs (×5000 magnification) showing the effects of (**a**) ATM, (**b**) **5**, and (**c**) a combination of ATM and **5** on cell morphology of NDM-1-producing *P. aeruginosa* (PA-017, *bla*_NDM_). The elongation of the bacterial cells by ATM was observed in a dose-dependent manner (**a**), but this morphological change was not evident with **5** (**b**). However, the addition of **5** to ATM promoted bacterial cell elongation, and significant cell elongation was observed even at a subinhibitory concentration of ATM (4 mg) (**c**).

To investigate the morphologic change of NDM-1-producing *P. aeruginosa* (PA-017, *bla*_NDM_) induced by antimicrobials, we observed PA-017 grown in different concentrations of ATM (Figure 4a), compound **5** (Figure 4b), and the combination of ATM and **5** (Figure 4c) by using a scanning electron microscope (SEM). The bacterial cells treated with increasing concentrations of ATM (0~16 mg/L, MIC = 16 mg/L) showed typical cell elongation (Figure 4a) due to selective inhibition of the penicillin-binding protein (PBP3), a protein known to be important to cell division [43]. This morphological change was not evident in the bacterial cells treated with **5** (Figure 4b). However, bacterial cell elongation was promoted when **5** was combined with ATM and, even at subinhibitory concentration of ATM (4 mg, 1/4 MIC), compound **5** increased cell elongation in a concentration-dependent manner (Figure 4c).

### 2.4. In Vivo Pharmacokinetic Study of ***5*** in Rats

**Figure 5:**
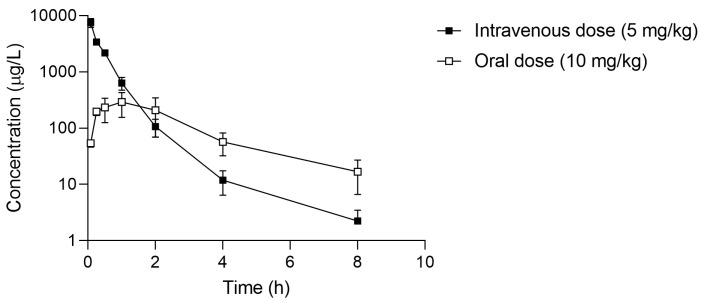
Mean concentration-time profiles of **5** in rats after administering single doses of 5 mg/kg intravenously (filled square) and 10 mg/kg orally (open square). Three male rats were used in each group. Data are presented as mean ± standard deviation.

The plasma concentration of **5** in rat plasma was determined after single intravenous administration of 5 mg/kg and oral administration of 10 mg/kg. The PK profile of the mean plasma concentration versus time is shown in Figure 5 and the PK parameters estimated are presented in Table 6.

The estimated mean half-life in plasma (*t*_1/2_) were 0.92 h and 1.28 h after intravenous (5 mg/kg) and oral (10 mg/kg) dose of **5**, respectively (Table 5). After intravenous administration, the mean maximum plasma concentration (C_max_), the area under the curve from time 0 to last time point (AUC_last_), the systemic clearance (Cl), and the volume of distribution at steady state (V_ss_) were observed as 7.51 mg/mL, 3.60 mg∙h/L, 23.55 mL/h/kg, and 0.65 mL/kg, respectively. On the other hand, after oral administration, the C_max_ and AUC_last_ were determined as 0.29 mg/L and 1.25 mg∙h/L, respectively, and both parameters are significantly increased compared with those of **1** (0.01 mg/L and 0.02 mg∙h/L, respectively) [36]. Accordingly, **5** showed higher mean oral bioavailability (F, 17.30%) compared with **1** (0.27%) [36].

### 2.5. In Vitro and In Vivo Toxicity of ***5***

**Figure 6:**
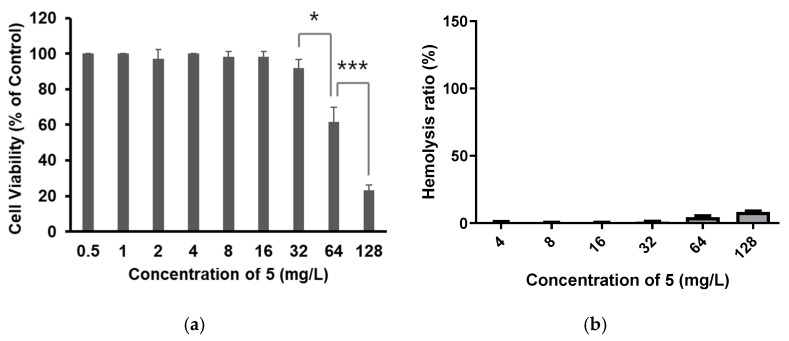
(**a**) Cytotoxicity of **5** (0.5–128 mg/L) on immobilized human embryonic kidney (HEK293) cells by the WST assay. (**b**) Human blood hemolysis assay for **5** (4–128 mg/L) using Triton X-100 and HEPES-NaOH buffered saline (HBS) as positive and negative controls, respectively. Data are expressed as mean ± SD. (*) *p* < 0.05 and (***) *p* < 0.001.

In vitro toxicity profile of **5** was assessed using cell viability and hemolysis tests. The cell viability evaluation of **5** in immortalized human embryonic kidney (HEK293) cells was performed by WST test. The results revealed that the viability was affected by **5** at concentrations greater than 64 mg/L (Figure 6a). In vitro hemolysis test was also performed using human red blood cells (RBCs), and **5** did not exhibit any noticeable toxic effect up to 128 mg/L (Figure 6b).

In vivo toxicity was also observed in 6-week-old C57BL/6J female mouse, and **5** did not show any toxicity up to 40 mg/kg.

## 3. Discussion

ATM’s natural tendency to resist hydrolysis by MBLs proposed a new strategy in a combat against MBL-producing Gram-negatives. However, ATM suffers from hydrolysis by several β-lactamases including AmpC, KPC, and NDM. In addition, due to the hyperactive efflux pumps, most MBL-positive *P. aeruginosa* strains remain resistant to ATM/AVI combination. Therefore, an inhibitor effective against the MBL-producing *P. aeruginosa* is required to be used in combination with ATM.

In this context, it is noteworthy that the 3-*O*-alkyl di-F-Q derivatives, multitarget inhibitors of broad-spectrum β-lactamases (including MBLs) and efflux pumps, revived ATM against MBL-producing *P. aeruginosa*. However, as the ATM-potentiating activity of the 3-*O*-alkyl di-F-Q derivatives was observed only at high and potentially toxic concentrations (32 mg/L), in this study, we attempted to enhance their ATM-potentiating activity through structural modification. Specifically, as both MBLs and efflux pumps reside in the periplasmic space, we intended porin-mediated influx of the inhibitors by making them polar and prepared a series of 3,7-bis-*O*-substituted di-F-Q derivatives through introduction of a polar substituent at the 7-OH position.

In general, the 7-*O*-substitutions on the 3-*O*-alkyl di-F-Q scaffold were accompanied by a loss of inhibitory activity against β-lactamases and efflux pumps. However, the α-aryloxy acetates (**5**~**7**) were identified as simultaneous inhibitors of β-lactamases and efflux pumps. The structure–activity relationship observed in this study indicates that the binding sites for the 3,7-bis-*O*-substituted di-F-Q derivatives in the β-lactamases and efflux pumps are short and narrow. Thus, the derivatives with relatively small and short 7-*O*-substituents such as acetates (**5**~**7**) and acetanilide (**9**) can show inhibitory activity against β-lactamases. In case of efflux pumps, the binding site looks even more limited to discriminate the acetates (**5**~**7**) and acetanilide (**9**) to result in selective inhibition of the efflux pumps by the acetates (**5**~**7**). The α-aryloxy acetates (**5**~**7**) were found to potentiate antimicrobial activity of ATM in a clinical strain of NDM-1-producing *P. aeruginosa* (PA-017, *bla*_NDM_). Of note, the derivatives **5**~7 reduced the MIC of ATM four- to eight-fold at concentrations (16 mg/L) even lower than those required by the previously reported 3-*O*-alkyl di-F-Q (32 mg/L).

The ATM-potentiating activity of **5** was further investigated in 26 clinical isolates of CPPAs and, in 22 out of 26 CPPA isolates (85%), ATM/**5** combination showed synergistic effects. Noteworthy is the fact that, in most cases, 4~16 mg/L of **5** was enough to show synergy with ATM. The combination of ATM (8 mg/L) and **5** (8 mg/L) inhibited 80% of the ATM-resistant CPPA, while ATM alone did not show any inhibition. More intriguingly, 8 mg/L ATM (clinical breakpoint) was enough to inhibit more than 80% of all the CPPA isolates (n = 26) and 15 ATM-resistant CPPA isolates by combination with 4 mg/L and 8 mg/L of **5**, respectively. In addition, only 4 mg/L of **5** was needed to reduce the MIC_90_ of ATM four-fold in ATM-resistant CPPA (n = 15). The time–kill data further supported the effectiveness of the combined treatment of ATM with **5**. Thus, combination of ATM (1xMIC) with 8 mg/L of **5** showed bactericidal effects in every bacterial strain tested (PA-002, *bla*_IMP_, PA-003, *bla*_VIM_, PA-014, *bla*_GES_, and PA-017, *bla*_NDM_) by reducing the bacterial loads by 5.1 log_10_~8.9 log_10_.

The in vivo PK data obtained in rats after oral (10 mg/kg) dose of **5** showed significant increases in C_max_, AUC_last_, and mean oral bioavailability (F) compared with **1**. Nevertheless, the cell-viability and hemolysis tests revealed no noticeable toxicity of **5** up to concentrations of 64 mg/L and 128 mg/L, respectively.

Our compounds have potential clinical implications. MBL-producing carbapenem-resistant *P. aeruginosa* is increasing worldwide, and infections caused by these organisms are associated with high mortality. However, to our knowledge, there are currently no US FDA-approved BL/BLi combinations effective against MBL-producing strains. Notably, the title compound **5** exhibited inhibitory activity against NDM-1, a representative MBL, as well as the efflux pump activity of *P. aeruginosa*, and the combined inhibitory activity resulted in synergistical potentiation of ATM. It should be noted that most carbapenem-resistant *P. aeruginosa* isolates tested were sensitized to 8 mg/L of ATM (clinical breakpoint) upon combination with 4~8 mg/L of **5**. New substances with inhibitory activity against MBL (taniborbactam, zidebactam, and xeruborbactam) are under research for FDA approval. Considering the rapid development of resistance due to the use of novel antimicrobials, diverse treatment strategies are necessary. Our dual inhibitors will be added to the arsenal against MBL-producing carbapenem-resistant *P. aeruginosa*.

This study has several limitations. First, our results in mice and rats cannot be extrapolated to humans. Comprehensive pharmacokinetic and pharmacodynamic studies in humans are necessary to confirm the compound’s clinical effectiveness and safety. Second, while the study discusses the inhibitory activity of compound **5** against NDM-1 and efflux pumps, it may not fully elucidate the mechanism of action, including potential off-target effects. Lastly, our study does not evaluate the potential for bacteria to develop resistance to the compound over time. Further research is needed to understand the compound’s impact on resistance patterns and its possible contribution to the emergence of more resistant strains.

## 4. Materials and Methods

### 4.1. Chemicals and Reagents

The reagents required for chemical synthesis were purchased from Sigma-Aldrich (St. Louis, MO, USA), TCI (Tokyo, Japan) or Alfa Aesar (Ward Hill, MA, USA). TLC and column chromatography were performed on silica gel-60 F254 and silica gel-60 (220–440 mesh) for flash chromatography, respectively, purchased from Merck (Rahway, NJ, USA). NMR spectra were recorded on JNM-ECZ500R (Tokyo, Japan) (500 MHz for ^1^H NMR and 125 MHz for ^13^C NMR). Chemical shifts (δ, ppm) were given relative to the solvent peak and multiplets were assigned as s (singlet), d (doublet), t (triplet), dd (doublet of doublet), td (triplet of doublet), ddd (doublet of doublet of doublet), m (multiplet), or br s (broad singlet) with the coupling constants reported in hertz (Hz). Fast atom bombardment (FAB) mass spectra (FAB-MS) were obtained at the Korea Basic Science Institute (Daegu, Republic of Korea) and reported in the form of *m*/*z* (intensity relative to the base peak = 100).

The reagents required for biological assay including dimethyl sulfoxide (DMSO), phosphate-buffered saline (PBS), glucose, ethidium bromide (EtBr), and carbonyl cyanide m-chlorophenyl hydrazine (CCCP) were purchased from Sigma-Aldrich. Cation-adjusted Mueller–Hinton broth (CAMHB) and Luria-Bertani (LB) media were obtained from BD Biosciences (Franklin Lakes, NJ, USA). Blood agar plates (BAP) and 4-(2-hydroxyethyl)-1-piperazineethanesulfonic acid (HEPES) were purchased from Hangang (Gunpo, Republic of Korea) and BioBasic (Markham, ON, Canada). Fetal bovine serum (FBS) and penicillin-streptomycin were purchased from Thermo Fisher Scientific (Waltham, MA, USA). TritonX-100, nitrocefin, and imipenem were obtained from Merck (Rahway, NJ, USA). Aztreonam was purchased from TCI (Tokyo, Japan).

### 4.2. Syntheses of the Title Compounds (***4***~***14***)

#### 4.2.1. Synthesis of 2-((3-(2-Cyclohexylethoxy)-2-(3,4-difluorophenyl)-5-hydroxy-4-oxo-4H-chromen-7-yl)oxy)succinic Acid (**4**)

To a solution of **3** (120 mg, 0.29 mmol) in CH_2_Cl_2_ (5 mL), di-*tert*-butyl but-2-ynedioate (68.8 μL, 0.32 mmol) and TEA (80.3 μL, 0.58 mmol) were added. After stirring at room temperature for 48 h, the reaction mixture was quenched with saturated aqueous NH_4_Cl solution and diluted with CH_2_Cl_2_. The organic layer was washed with brine, dried over MgSO_4_, filtered, and concentrated under reduced pressure. The residue was purified by column chromatography on silica gel (Hexanes:Et_2_O = 10:1) to afford the desired adduct, di-*tert*-butyl 2-aryloxymaleate, as a yellow solid. The degassed suspension of the adduct obtained above (110 mg, 0.17 mmol) and 10% Pd/C (11 mg) in MeOH (3 mL) was vigorously stirred under an atmosphere of H_2_ (balloon) for 1 h. The reaction mixture was filtered through a short Celite pad and concentrated under reduced pressure. The residue was purified by column chromatography on silica gel (Hexanes:Et_2_O = 10:1) to afford the reduced product, di-*tert*-butyl 2-aryloxysuccinate, as a yellow solid. To a solution of the product obtained above (98 mg, 0.15 mmol) in CH_2_Cl_2_ (3 mL) TFA was added (0.3 mL), and the resulting mixture was stirred at room temperature for 48 h. The reaction mixture was then concentrated under reduced pressure and the residue was recrystallized with MeOH and CH_2_Cl_2_ to afford **4** (27 mg, 0.05 mmol, 33% yield) as a yellow solid: ^1^H NMR (500 MHz, methanol-*d*_4_) δ 8.03 (ddd, *J* = 2.2, 7.8, 12.0 Hz, 1H), 7.93–7.90 (m, 1H), 7.45 (ddd, *J* = 8.6, 8.6, 10.3 Hz, 1H), 6.63 (d, *J* = 2.3 Hz, 1H), 6.35 (d, *J* = 2.3 Hz, 1H), 5.29 (dd, *J* = 3.7, 8.4 Hz, 1H), 4.07 (t, *J* = 6.5 Hz, 2H), 3.05 (dd, *J* = 3.7, 16.7 Hz, 1H), 2.95 (dd, *J* = 8.4, 16.7 Hz, 1H), 1.70–1.63 (m, 5H), 1.56 (q, *J* = 6.7 Hz, 2H), 1.44–1.35 (m, 1H), 1.22–1.13 (m, 3H), 0.92–0.84 (m, 2H); ^13^C NMR (125 MHz, methanol-*d*_4_) δ 180.2, 172.9, 172.8, 165.4, 162.9, 158.0, 155.2, 152.9 (dd, *J* = 12.7, 251.7 Hz), 151.2 (dd, *J* = 12.8, 245.4 Hz), 140.1, 129.0–128.9 (m), 127.0 (dd, *J* = 3.5, 7.0 Hz), 119.2 (d, *J* = 19.9 Hz), 118.7 (d, *J* = 17.8 Hz), 107.4, 100.0, 94.7, 74.5, 72.0, 38.5, 38.2, 35.4, 34.3, 27.6, 27.3; FAB-MS calcd for C_27_H_27_F_2_O_9_ [M + H]^+^ 533.2, found 533.5.

#### 4.2.2. General Procedure A for Preparation of **5**~**7**

##### General Procedure A-1. Preparation of the Ethyl 2-Aryloxyacetates from **1**~**3**

A solution of **1**~**3** (1.26 mmol) in DMF (10 mL) was added to ethyl bromoacetate (1.39 mmol) and K_2_CO_3_ (1.89 mmol). After stirring at 50 °C for 6 h, the reaction mixture was cooled to room temperature and diluted with EtOAc. The reaction was quenched with saturated aq. NH_4_Cl, and the organic layer was washed with brine, dried over MgSO_4_, and filtered. The filtrate was concentrated under reduce pressure, and the crude product was purified by column chromatography on silica gel (Hex:CH_2_Cl_2_:EtOAc = 6:1:1) to afford the corresponding ethyl 2-aryloxyacetate (0.83 mmol, 66% yield) as a yellow solid.

##### General Procedure A-2. Preparation of **5**~**7** from the Ethyl 2-Aryloxyacetates

The ethyl 2-aryloxyacetate obtained above (0.42 mmol) was dissolved in a mixture of THF (3 mL) and MeOH (3 mL) and treated with aq. 2 N NaOH (3 mL). The resulting mixture was stirred at room temperature for 2 h, acidified with aq. 2 N HCl, and extracted with EtOAc. The combined organic layers were washed with brine, dried over MgSO_4_, and concentrated under reduced pressure. The residue was triturated with diethyl ether to afford the desired product **5**~**7**.

#### 4.2.3. Synthesis of 2-((2-(3,4-Difluorophenyl)-3-(3,3-dimethylbutoxy)-5-hydroxy-4-oxo-4H-chromen-7-yl)oxy)acetic Acid (**5**)

General procedures A-1 and A-2 were adopted using **1** as a starting material, and the desired product **5** was obtained as a yellow solid with 83% yield: ^1^H NMR (500 MHz, methanol-*d*_4_) δ 8.05 (ddd, *J* = 2.2, 7.8, 12.1 Hz, 1H), 7.97–7.94 (m, 1H), 7.45 (ddd, *J* = 8.6, 8.6, 10.3 Hz, 1H), 6.60 (d, *J* = 2.3 Hz, 1H), 6.35 (d, *J* = 2.3 Hz, 1H), 4.77 (s, 2H), 4.10 (t, *J* = 7.5 Hz, 2H), 1.68 (t, *J* = 7.5 Hz, 2H), 0.94 (s, 9H); ^13^C NMR (125 MHz, DMSO-*d*_6_) δ 178.4, 169.5, 164.0, 160.9, 156.3, 153.2, 150.8 (dd, *J* = 12.3, 250.6 Hz), 149.3 (dd, *J* = 12.8, 244.3 Hz), 138.6, 127.6–127.5 (m), 126.1–126.0 (m), 118.1 (d, *J* = 17.2 Hz), 117.7 (d, *J* = 19.5 Hz), 105.7, 98.5, 93.2, 70.2, 65.1, 42.7, 29.5, 29.5; FAB-MS calcd for C_23_H_23_F_2_O_7_ [M + H]^+^ 449.1, found 449.4.

#### 4.2.4. Synthesis of 2-((3-(2-Cyclopentylethoxy)-2-(3,4-difluorophenyl)-5-hydroxy-4-oxo-4H-chromen-7-yl)oxy)acetic Acid (**6**)

General procedures A-1 and A-2 were adopted using **2** as a starting material, and the desired product **6** was obtained as a yellow solid with 49% yield: ^1^H NMR (500 MHz, acetone-*d*_6_) δ 8.13 (ddd, *J* = 2.2, 8.0, 12.2 Hz, 1H), 8.07–8.04 (m, 1H), 7.57 (ddd, *J* = 8.6, 8.6, 10.3 Hz, 1H), 6.75 (d, *J* = 2.3 Hz, 1H), 6.40 (d, *J* = 2.3 Hz, 1H), 4.91 (s, 2H), 4.15 (t, *J* = 6.7 Hz, 2H), 1.97–1.89 (m, 1H), 1.79–1.73 (m, 4H), 1.64–1.56 (m, 2H), 1.55–1.46 (m, 2H), 1.14–1.07 (m, 2H); ^13^C NMR (125 MHz, acetone-*d*_6_) δ 179.8, 169.3, 165.1, 162.6, 157.6, 154.4, 152.3 (dd, *J* = 12.6, 250.8 Hz), 150.7 (dd, *J* = 13.1, 244.7 Hz), 139.9, 129.0–128.9 (m), 126.9 (dd, *J* = 3.8, 7.1 Hz), 118.7 (d, *J* = 20.6 Hz), 118.6 (d, *J* = 17.9 Hz), 107.1, 99.1, 93.9, 73.0, 65.6, 37.4, 36.8, 33.2, 25.6; FAB-MS calcd for C_24_H_23_F_2_O_7_ [M + H]^+^ 461.1, found 461.4.

#### 4.2.5. Synthesis of 2-((3-(2-Cyclohexylethoxy)-2-(3,4-difluorophenyl)-5-hydroxy-4-oxo-4H-chromen-7-yl)oxy)acetic Acid (**7**)

General procedures A-1 and A-2 were adopted using **3** as a starting material, and the desired product **7** was obtained as a yellow solid with 50% yield: ^1^H NMR (500 MHz, acetone-*d*_6_) δ 8.11 (ddd, *J* = 2.2, 8.0, 12.0 Hz, 1H), 8.04–8.01 (m, 1H), 7.56 (ddd, *J* = 8.7, 8.7, 10.3 Hz, 1H), 6.73 (d, *J* = 2.3 Hz, 1H), 6.38 (d, *J* = 2.3 Hz, 1H), 4.89 (s, 2H), 4.18 (t, *J* = 6.6 Hz, 2H), 1.71–1.64 (m, 5H), 1.60 (td, *J* = 6.6, 6.7 Hz, 2H), 1.48–1.39 (m, 1H), 1.25–1.12 (m, 3H), 0.94–0.86 (m, 2H); ^13^C NMR (125 MHz, acetone-*d*_6_) δ 179.8, 169.3, 165.1, 162.6, 157.6, 154.4, 152.3 (dd, *J* = 12.7, 250.9 Hz), 150.7 (dd, *J* = 12.8, 244.5 Hz), 139.9, 129.0–128.9 (m), 126.9 (dd, *J* = 3.7, 7.1 Hz), 118.8 (d, *J* = 19.9 Hz), 118.6 (d, *J* = 17.5 Hz), 107.1, 99.1, 93.9, 71.4, 65.6, 38.1, 35.0, 33.9, 27.2, 26.9; FAB-MS calcd for C_25_H_25_F_2_O_7_ [M + H]^+^ 475.2, found 475.4.

#### 4.2.6. Synthesis of 2-((2-(2-((3-(2-Cyclohexylethoxy)-2-(3,4-difluorophenyl)-5-hydroxy-4-oxo-4H-chromen-7-yl)oxy)acetamido)ethyl)dimethylammonio)acetate (**8**)

General procedure A-1 was adopted using **1** (1.26 mmol) as a starting material, and the corresponding ethyl 2-aryloxyacetate (0.83 mmol, 66% yield) was obtained as a yellow solid. The product obtained above (252 mg, 0.50 mmol) was dissolved in EtOH (5 mL) and treated with *N*,*N*-dimethyl ethylenediamine (137 μL, 1.25 mmol). The resulting mixture was stirred under reflux for 12 h and, after cooling to room temperature, concentrated under reduced pressure. The residue was triturated with diethyl ether to afford the corresponding adduct as off-white solid. To a solution of the adduct obtained above (87 mg, 0.16 mmol) in DMF (3 mL) *tert*-butyl 2-bromoacetate was added (47 μL, 0.32 mmol), and the resulting mixture was stirred at room temperature for 12 h. The precipitate was filtered and washed with EtOAc to afford the *tert*-butyl acetate as a yellow solid. The product obtained above (68 mg) was treated with 4 M HCl in 1,4-dioxane (3 mL) and stirred at room temperature for 3 h. The reaction mixture was concentrated under reduced pressure and the crude product was triturated with EtOAc and acetone to afford **8** (31 mg, 0.05 mmol, 56% yield) as a yellow solid: ^1^H NMR (500 MHz, methanol-*d*_4_) δ 7.99 (ddd, *J* = 2.2, 7.8, 11.9 Hz, 1H), 7.91–7.88 (m, 1H), 7.43 (ddd, *J* = 8.5, 8.5, 10.3 Hz, 1H), 6.66 (d, *J* = 2.3 Hz, 1H), 6.42 (d, *J* = 2.3 Hz, 1H), 4.63 (s, 2H), 4.34 (s, 2H), 4.04 (t, *J* = 6.5 Hz, 2H), 3.82–3.75 (m, 4H), 3.34 (s, 6H), 1.68–1.61 (m, 5H), 1.53 (td, *J* = 6.5, 6.6 Hz, 2H), 1.41–1.33 (m, 1H), 1.22–1.08 (m, 3H), 0.89–0.82 (m, 2H); ^13^C NMR (125 MHz, methanol-*d*_4_) δ 180.3, 170.8, 167.2, 164.9, 163.1, 158.1, 155.3, 153.0 (dd, *J* = 12.5, 251.8 Hz), 151.3 (dd, *J* = 13.1, 246.0 Hz), 140.3, 129.0–128.9 (m), 127.1–127.0 (m), 119.2 (d, *J* = 20.1 Hz), 118.8 (d, *J* = 17.9 Hz), 107.7, 99.8, 94.2, 72.1, 68.3, 63.9, 62.3, 52.9, 38.6, 35.4, 34.5, 34.3, 27.6, 24.4; FAB-MS calcd for C_31_H_37_F_2_N_2_O**_8_** [M + H]^+^ 603.2, found 603.6.

#### 4.2.7. General Procedure B for Preparation of **9**~**11**

To a solution of **5** (100 mg, 0.22 mmol) in CH_2_Cl_2_ (5 mL), amine derivatives (0.24 mmol), DMAP (54.5 mg, 0.45 mmol) and EDC·HCl (85.5 mg, 0.45 mmol) were added, and the resulting mixture was stirred overnight at room temperature. After concentration under reduced pressure, the residue was purified by column chromatography on silica gel to give the desired product.

#### 4.2.8. Synthesis of 2-((2-(3,4-Difluorophenyl)-3-(3,3-dimethylbutoxy)-5-hydroxy-4-oxo-4H-chromen-7-yl)oxy)-N-phenylacetamide (**9**)

General procedure B was adopted using aniline as an amidating agent, and the crude product was purified by column chromatography on silica gel (Hex:EtOAc = 5:1) to afford **9** (90 mg, 0.17 mmol, 77% yield) as a yellow solid:^1^H NMR (500 MHz, DMSO-*d*_6_) δ 12.48 (s, 1H), 10.15 (s, 1H), 8.11 (ddd, *J* = 2.1, 7.9, 12.1 Hz, 1H), 7.98–7.95 (m, 1H), 7.70 (ddd, *J* = 8.7, 8.7, 10.4 Hz, 1H), 7.64–7.62 (m, 2H), 7.35–7.31 (m, 2H), 7.11–7.07 (m, 1H), 6.87 (d, *J* = 2.2 Hz, 1H), 6.51 (d, *J* = 2.2 Hz, 1H), 4.87 (s, 2H), 4.10 (t, *J* = 7.5 Hz, 2H), 1.62 (t, *J* = 7.5 Hz, 2H), 0.90 (s, 9H) ^13^C NMR (125 MHz, DMSO-*d*_6_) δ 178.4, 165.6, 164.0, 161.0, 156.3, 153.4, 150.8 (dd, *J* = 12.3, 251.4 Hz), 149.3 (dd, *J* = 12.8, 244.5 Hz), 138.6, 138.3, 128.9, 127.6–127.5 (m), 126.1–126.0 (m), 123.9, 119.7, 118.2 (d, *J* = 17.5 Hz), 117.7 (d, *J* = 19.5 Hz), 105.9, 98.6, 93.5, 70.2, 67.3, 42.7, 29.5, 29.5; FAB-MS calcd for C_29_H_28_F_2_NO_6_ [M + H]^+^ 524.2, found 524.5.

#### 4.2.9. Synthesis of 2-(3,4-Difluorophenyl)-3-(3,3-dimethylbutoxy)-5-hydroxy-7-(2-oxo-2-(piperidin-1-yl)ethoxy)-4H-chromen-4-one (**10**)

General procedure B was adopted using piperidine as an amidating agent, and the crude product was purified by column chromatography on silica gel (Hex:EtOAc = 3:2) to afford **10** (91 mg, 0.18 mmol, 79% yield) as a yellow solid: ^1^H NMR (500 MHz, DMSO-*d*_6_) δ 12.46 (s, 1H), 8.10 (ddd, *J* = 2.2, 7.9, 12.1 Hz, 1H), 7.97–7.94 (m, 1H), 7.70 (ddd, *J* = 8.7, 8.7, 10.5 Hz, 1H), 6.78 (d, *J* = 2.2 Hz, 1H), 6.41 (d, *J* = 2.2 Hz, 1H), 4.98 (s, 2H), 4.09 (t, *J* = 7.5 Hz, 2H), 3.44–3.41 (m, 2H), 3.39–3.37 (m, 2H), 1.63–1.52 (m, 4H), 1.61 (t, *J* = 7.5 Hz, 2H), 1.48–1.41 (m, 2H), 0.89 (s, 9H); ^13^C NMR (125 MHz, CDCl_3_) δ 178.9, 164.9, 164.0, 162.1, 156.6, 153.5, 151.7 (dd, *J* = 12.6, 253.5 Hz), 150.1 (dd, *J* = 12.7, 247.0 Hz), 139.2, 127.5–127.4 (m), 125.3 (dd, *J* = 3.8, 6.7 Hz), 118.0 (d, *J* = 19.0 Hz), 117.5 (d, *J* = 17.6 Hz), 106.6, 98.7, 92.8, 71.0, 67.5, 46.4, 43.3, 43.3, 29.7, 29.6, 26.5, 25.5, 24.4; FAB-MS calcd for C_28_H_32_F_2_NO_6_ [M + H]^+^ 516.2, found 516.5.

#### 4.2.10. Synthesis of (S)-6-Amino-2-(2-((2-(3,4-difluorophenyl)-3-(3,3-dimethylbutoxy)-5-hydroxy-4-oxo-4H-chromen-7-yl)oxy)acetamido)hexanoic Acid Hydrochloride (**11**)

General procedure B was adopted using H-Lys(Boc)-OtBu·HCl as an amidating agent, and the crude product was purified by column chromatography on silica gel (CH_2_Cl_2_:MeOH = 50:1) to afford a protected lysine adduct (62% yield) as a yellow solid. To a cooled solution of the product obtained above (100 mg, 0.14 mmol) in CH_2_Cl_2_ (3 mL), TFA was added (3 mL), and the resulting mixture was stirred at room temperature for 12 h. The reaction mixture was then concentrated under reduced pressure, and the residue was taken by diethyl ether containing 2N HCl. The precipitate was filtered and washed with acetone to afford **11** (36 mg, 0.06 mmol, 43% yield) as a yellow solid: ^1^H NMR (500 MHz, methanol-*d*_4_) δ 8.05 (ddd, *J* = 2.2, 7.8, 12.0 Hz, 1H), 7.98–7.95 (m, 1H), 7.46 (ddd, *J* = 8.6, 8.6, 10.3 Hz, 1H), 6.68 (d, *J* = 2.3 Hz, 1H), 6.43 (d, *J* = 2.3 Hz, 1H), 4.71 (s, 2H), 4.53 (dd, *J* = 4.9, 9.1 Hz, 1H), 4.10 (t, *J* = 7.4 Hz, 2H), 2.97–2.88 (m, 2H), 2.04–1.97 (m, 1H), 1.86–1.80 (m, 1H), 1.77–1.64 (m, 2H), 1.68 (t, *J* = 7.5 Hz, 2H), 1.56–1.44 (m, 2H), 0.94 (s, 9H); ^13^C NMR (125 MHz, methanol-*d*_4_) δ 180.3, 174.8, 170.1, 165.2, 163.0, 158.1, 155.2, 152.9 (dd, *J* = 12.6, 240.7 Hz), 151.3 (dd, *J* = 13.0, 239.7 Hz), 140.3, 129.1–129.0 (m), 127.0–126.9 (m), 119.0 (d, *J* = 17.9 Hz), 118.8 (d, *J* = 18.0 Hz), 107.6, 99.7, 94.4, 71.9, 68.2, 53.0, 44.4, 40.5, 32.0, 30.5, 30.0, 28.0, 23.8; FAB-MS calcd for C_29_H_35_F_2_N_2_O_8_ [M + H]^+^ 577.2, found 577.6.

#### 4.2.11. General Procedure C for Preparation of **12**~**14**

To a cooled solution of **1** or **2** (0.26 mmol) in THF (5 mL), an appropriate alcohol (0.28 mmol), PPh_3_ (0.51 mmol), and DIAD (0.51 mmol) were added, and the resulting mixture was stirred for 1 h. The reaction mixture was concentrated under reduced pressure and the residue was purified by column chromatography in silica gel to give the desired product.

#### 4.2.12. Synthesis of 2-(3,4-Difluorophenyl)-3-(3,3-dimethylbutoxy)-5-hydroxy-7-(2-(1-methylpyrrolidin-2-yl)ethoxy)-4H-chromen-4-one (**12**)

General procedure C was adopted using **1** as a starting material and 1-methyl-2-pyrrolidineethanol as an alkylating agent, and the crude product was purified by column chromatography on silica gel (CH_2_Cl_2_:MeOH:aq. NH_4_OH = 100:10:1) to afford **12** (55% yield) as a yellow solid: ^1^H NMR (500 MHz, methanol-*d*_4_) δ 8.06 (ddd, *J* = 2.2, 7.8, 12.0 Hz, 1H), 8.00–7.97 (m, 1H), 7.48 (ddd, *J* = 8.6, 8.6, 10.2 Hz, 1H), 6.70 (d, *J* = 2.2 Hz, 1H), 6.41 (d, *J* = 2.3 Hz, 1H), 4.31 (m, 2H), 4.12 (t, *J* = 7.5 Hz, 2H), 3.70–3.63 (m, 1H), 3.58–3.51 (m, 1H), 3.24–3.15 (m, 1H), 2.96 (s, 3H), 2.53–2.40 (m, 2H), 2.20–2.03 (m, 3H), 1.94–1.86 (m, 1H), 1.69 (t, *J* = 7.5 Hz, 2H), 0.94 (s, 9H); ^13^C NMR (125 MHz, DMSO-*d*_6_) δ 178.4, 164.3, 161.0, 156.4, 153.2, 150.8 (dd, *J* = 12.5, 238.7 Hz), 149.3 (dd, *J* = 12.4, 244.8 Hz), 138.6, 127.6–127.5 (m), 126.1–126.0 (m), 118.2 (d, *J* = 17.4 Hz), 117.7 (d, *J* = 19.6 Hz), 105.7, 98.4 93.2, 70.2, 65.8, 65.5, 57.1, 55.1, 42.7, 32.6, 29.8, 29.5, 29.5, 21.1; FAB-MS calcd for C_28_H_34_F_2_NO_5_ [M + H]^+^ 502.2, found 502.6.

#### 4.2.13. Synthesis of 2-(3,4-Difluorophenyl)-3-(3,3-dimethylbutoxy)-5-hydroxy-7-(2-(4-methylpiperazin-1-yl)ethoxy)-4H-chromen-4-one (**13**)

General procedure C was adopted using **1** as a starting material and 1-methylpiperazine as an alkylating agent, and the crude product was purified by column chromatography on silica gel (CH_2_Cl_2_:MeOH:aq. NH_4_OH = 100:10:1) to afford **13** (29% yield) as a yellow oil: ^1^H NMR (500 MHz, CDCl_3_) δ 12.51 (s, 1H), 7.99 (ddd, *J* = 2.2, 7.8, 11.8 Hz, 1H), 7.92–7.89 (m, 1H), 7.30 (ddd, *J* = 8.6, 8.6, 9.7 Hz, 1H), 6.45 (d, *J* = 2.2 Hz, 1H), 6.36 (d, *J* = 2.2 Hz, 1H), 4.17 (t, *J* = 5.8 Hz, 2H), 4.11 (t, *J* = 7.8 Hz, 2H), 2.85 (t, *J* = 5.8 Hz, 2H), 2.63 (br s, 4H), 2.49 (br s, 4H), 2.31 (s, 3H), 1.70 (t, *J* = 7.9 Hz, 2H), 0.93 (s, 9H); ^13^C NMR (125 MHz, CDCl_3_) δ 178.9, 164.8, 162.1, 156.6, 153.2, 151.6 (dd, *J* = 12.6, 253.6 Hz), 150.1 (dd, *J* = 12.8, 246.8 Hz), 139.2, 127.6–127.6 (m), 125.3–125.2 (m), 117.9 (d, *J* = 20.5 Hz), 117.5 (d, *J* = 17.4 Hz), 106.2, 98.5, 92.7, 71.0, 66.7, 56.7, 55.0, 53.5, 46.0, 43.3, 29.7, 29.6; FAB-MS calcd for C_28_H_35_F_2_N_2_O_5_ [M + H]^+^ 517.2, found 517.6.

#### 4.2.14. Synthesis of 3-(2-Cyclopentylethoxy)-2-(3,4-difluorophenyl)-5-hydroxy-7-(2-(4-methylpiperazin-1-yl)ethoxy)-4H-chromen-4-one (**14**)

General procedure C was adopted using **2** as a starting material and 1-methylpiperazine as an alkylating agent, and the crude product was purified by column chromatography on silica gel (CH_2_Cl_2_:MeOH:aq. NH_4_OH = 100:10:1) to afford **14** (34% yield) as a yellow oil: ^1^H NMR (500 MHz, CDCl_3_) δ 12.50 (s, 1H), 7.99 (ddd, *J* = 2.2, 7.7, 11.7 Hz, 1H), 7.91–7.87 (m, 1H), 7.29 (ddd, *J* = 8.7, 8.7, 9.9 Hz, 1H), 6.45 (d, *J* = 2.2 Hz, 1H), 6.36 (d, *J* = 2.2 Hz, 1H), 4.17 (t, *J* = 5.8 Hz, 2H), 4.06 (t, *J* = 7.0 Hz, 2H), 2.85 (t, *J* = 5.8 Hz, 2H), 2.63 (br s, 4H), 2.48 (br s, 4H), 2.30 (s, 3H), 1.93–1.84 (m, 1H), 1.77–1.71 (m, 2H), 1.74 (td, *J* = 7.0, 7.1 Hz, 2H), 1.64–1.56 (m, 2H), 1.53–1.47 (m, 2H), 1.12–1.05 (m, 2H); ^13^C NMR (125 MHz, CDCl_3_) δ 178.9, 164.8, 162.1, 156.6, 153.4, 151.6 (dd, *J* = 12.9, 253.5 Hz), 150.1 (dd, *J* = 12.6, 246.9 Hz), 139.1, 127.6–127.5 (m), 125.3 (dd, *J* = 3.7, 6.6 Hz), 118.0 (d, *J* = 19.4 Hz), 117.5 (d, *J* = 17.5 Hz), 106.2, 98.5, 92.8, 72.8, 66.7, 56.7, 55.0, 53.5, 46.0, 36.6, 36.2, 32.6, 25.0; FAB-MS calcd for C_29_H_35_F_2_N_2_O_5_ [M + H]^+^ 529.2, found 529.6.

### 4.3. Microorganisms

Twenty-six carbapenemase-producing *P. aeruginosa* clinical isolates (*bla*_GES_, *bla*_VIM_, *bla*_IMP_, *bla*_NDM_, *bla*_IMP/NDM_) were collected from patients admitted to a university hospital in Seoul, Republic of Korea.

### 4.4. Inhibition of β-Lactamase Activity

Recombinant AmpC, OXA-48 (residues 23–265) and VIM-2 (residues 27–266) were prepared from *Escherichia coli* based on previously established protocols [36]. KPC-2 and NDM-1 are commercially available from LifeSpan Biosciences, Inc., Seattle, WA, USA (LS-G26598) and RayBiotech, Inc., Peachtree Corners, GA, USA (230-00554), respectively. For β-lactamase inhibition assay, in a 96-well plate, 70–0.1 μM (in PBS) of the synthesized compounds (**1**~**14**) (100 μL) were mixed with diluted β-lactamase stock (2% DMSO in 200 μL). In a separate 96-well plate, a substrate of AmpC, KPC-2, OXA-48, and VIM-2 (nitrocefin) and NDM-1 (imipenem) were added into each well (final concentrations of 70 μM for nitrocefin and 100 μM for imipenem). After incubation at 37 °C for 20 min, the mixture of the synthesized compounds (**1**~**14**) and β-lactamase in one plate was added to the substrate in the other plate. Enzymatic hydrolysis of the substrate was then monitored by a Cytation 5 imaging multimode reader (BioTek Instruments Inc., Winooski, VT, USA) at 486 nm and 300 nm for nitrocefin and imipenem, respectively, for 15 min. The rates of hydrolysis in the presence of the inhibitors were determined when the reaction rate plateaued. Comparison of the enzymatic hydrolysis rates in the presence and the absence of inhibitors (100%) provided the percentage of enzyme inhibition at each concentration of the synthesized compounds. The IC_50_ values were obtained after non-linear regression (sigmoidal dose-responses) by GraphPad Prism 5 (GraphPad Software, Boston, MA, USA).

### 4.5. Inhibition of Efflux Pump Activity

The NDM-producing *P. aeruginosa* clinical isolate (PA-017, *bla*_NDM_) (5 mL, LB medium) was grown to reach the mid-log phase. After centrifugation at 3500 rpm for 15 min, the bacterial pellet was washed twice with PBS and resuspended to OD_600_ = 0.3 in PBS. To each well of a flat-bottomed black 96-well plate, 182 µL of the bacterial suspension, 4 µL of glucose [final concentration = 0.4% (*w*/*v*)], 4 µL of the synthesized compounds or positive control CCCP, and 10 µL of EtBr (final concentration = 1 µg/mL) were added. The plate was incubated at 37 °C, and fluorescence emission from EtBr (λ_ex_ 480 nm/λ_em_ 630 nm) was recorded every 2 min (for 60 min) using a Cytation 5 imaging multi-mode reader (BioTek Instruments, Inc., Winooski, VT, USA).

### 4.6. Antimicrobial Susceptibility Assay

According to the Clinical and Laboratory Standards Institute (CLSI) guidelines [44], the minimum inhibitory concentration (MIC) of antibiotics and the synthesized compounds (**1**–**14**) was determined for bacteria of interest in three replicates using broth microdilution with CAMHB. The serially diluted DMSO stock solutions of the antimicrobial agents (190 μL in CAMHB) were mixed with 10 μL of the bacterial suspension (5 × 10^5^ CFU/mL) in 96-well microtiter plates (1% DMSO, final). The plate was incubated at 37 °C for 24 h, and the turbidity of each well was visually evaluated to determine bacterial growth. The lowest concentrations of the antimicrobial agents inhibiting the growth of the bacteria were determined as MICs.

### 4.7. Checkerboard Assay

Sterile CAMHB (100 μL) was dispensed into clear sterile 96-well microplates and inoculated with overnight culture of each strain to give a final inoculum of 5 × 10^5^ CFU/mL. Samples of the test compounds (synthesized compounds) and ATM were prepared in varying concentrations (0.06–512 μg/mL) using the two-fold serial dilution method and added to each well of the 96-well microplate containing bacterial in horizontal and vertical directions, respectively, to make a total volume of 200 μL of broth. After incubation at 37 °C for 18–24 h, the microplate was visually inspected to determine MICs of ATM combined with the test compounds. The determined MICs of ATM alone (MIC_ATM_), the test compound alone (MIC_test_), ATM combined with the test compound (MIC_ATM+test_), and the test compound combined with ATM (MIC_test+ATM_) were used to calculate the fractional inhibitory concentration index (FICI) as follows:

FICI = MIC_ATM+test_/MIC_ATM_ + MIC_test+ATM_/MIC_test_ (MIC_test_ = 256 mg/L)

The combinations of ATM and the test compounds were determined as synergistic (FICI ≤ 0.5), additive (0.5 < FICI ≤ 1.0), indifferent (1.0 < FICI ≤ 4.0), or antagonistic (FICI > 4.0) according to the FICI values.

### 4.8. Time–Kill Assay

NDM-producing *P. aeruginosa* (PA-017, *bla*_NDM_; 10^6^ CFU/mL) prepared from exponentially growing cells were placed into conical tubes and incubated in the presence of ATM alone (1/4 MIC, 1/2 MIC, 1 MIC, and 2 MIC) and in combination with the compound **5** (8 mg/L) in a final volume of 2 mL at 37 °C. Aliquots (50 μL) were taken from the incubation mixture at regular time intervals (t = 0, 3, 6, and 24 h), serially diluted in 0.85% NaCl solution, and plated on BAP. Bacterial colonies were counted, and the data are presented as the means of at least three independent experiments.

### 4.9. In Vivo Pharmacokinetics Study

After administration of compound **5** (5 mg/kg intravenously and 10 mg/kg orally) to specific pathogen-free (SPF) male Sprague–Dawley (SD) rats (Medicilon Colony: 999M-017), samples of serum were taken at eight different time points (0.083, 0.25, 0.5, 1, 2, 4, 8, and 24 h). Analysis of the samples were performed by liquid chromatography with tandem mass spectrometry (LC-MS/MS). A standard set of parameters were calculated using non-compartmental analysis modules in FDA certified pharmacokinetic program Phoenix WinNonlin 7.0 (Pharsight, Mountain View, CA, USA).

### 4.10. Field-Emission Scanning Electron Microscope Analysis

The morphology of *P. aeruginosa* cells treated with ATM and/or compound **5** was visualized using a field-emission scanning electron microscope (FESEM) as previously described by us with some modifications [45]. Bacterial suspensions with varying concentrations of ATM, compound **5**, and both were prepared in 24-well plates pre-placed with sterile cover glass slips. The samples were incubated at 37 °C for 24 h and then fixed with 2.5% glutaraldehyde overnight at 4 °C. Bacterial cells underwent three 10-min washes with 0.1 M sodium cacodylate buffer. The samples were dehydrated with increasing concentrations of ethanol (15%, 30%, 50%, 70%, and 80%) for 15 min each. Bacterial cells were incubated with 95% ethanol for 30 min and then with 100% ethanol twice for 30 min each. The samples were cross-linked with a 1:1 mixture of hexamethyldisilazane (HMDS) and 100% ethanol for 15 min, followed by two rounds of HMDS alone for 15 min each. After removing HMDS, the samples were dried overnight in a glass desiccator containing silica gel. Coverslips were mounted on stubs and coated with gold particles using an E-1045 ION sputter coater. Images were captured with a FESEM (S-4800; Hitachi, Tokyo, Japan).

### 4.11. Toxicity Assessment (1): Cytotoxicity Assay

The HEK293 cells were cultured in Dulbecco’s modified Eagle medium (DMEM) supplemented with 10% fetal bovine serum (FBS) and 1% penicillin-streptomycin solution at 37 °C with 5% CO_2_. The cells seeded into 96-well plates at 1.5 × 10^4^ cells/well were incubated for 24 h. The compound **5** was added to the wells at concentrations ranging from 0.5 to 128 mg/L and incubated for 24 h. Then, for WST-1 assay, EZ-cytox solution (10 μL, DoGenBio, Seoul, Republic of Korea) was added to the wells and incubated for 1 h, and the absorbance was measured at 450 nm (Cytation 5 imaging multimode reader, BioTek Instruments, Inc., Winooski, VT, USA).

### 4.12. Toxicity Assessment (2): Hemolytic Activity Assay

Washed human red blood cells (hRBCs, Innovative Research, Novi, MI, USA) were diluted 1:100 in HEPES-NaOH buffered saline (HBS) [150 mM NaCl/5 mM HEPES-NaOH (pH 7.4)]. Samples (100 μL) were taken from the serially diluted (4 to 128 mg/L) compound **5** and mixed with 250 μL of hRBCs in 1.5 mL microcentrifuge tube. After incubation at 37 °C for 30 min, the tubes were centrifuged at 3000 rpm for 5 min. The supernatant (50 μL), thus obtained, was transferred to transparent, flat-bottom 96-well plates and absorption was measured at 405 nm (Cytation 5 imaging multimode reader, BioTek Instruments, Inc., Winooski, VT, USA). In some cases, 1% TritonX-100 or HBS buffer was used instead of the compound **5** as positive and negative controls, respectively. The percentage of hemolysis was calculated as follows: % Hemolysis = (OD_test_ − OD_HBS_)/(OD_TritonX_/OD_HBS_) × 100%.

## Figures and Tables

**Table 1:** Physicochemical properties and inhibitory activity of compounds **1**~**14** against various β-lactamases and efflux pumps. ATM-potentiating activity of the compounds **1**~**14** in NDM-producing clinical strain of *P. aeruginosa* (PA-017, *bla*_NDM_) are also shown.

Cmpd	Net Charge	cLogD_7.4_	PSA (Å^2^)	BLi, IC_50_ (μM)					NDM-1-Producing *P. aeruginosa* (PA-017, *bla*_NDM_)				
				AmpC	KPC-2	OXA-48	VIM-2	NDM-1	EPi *^a^*	MIC (mg/L) *^b^*			
										**ATM**	**Cmpd**	**ATM + Cmpd *^c^***	**FD *^d^***
**1**	0	2.56	76.0	18.9 ± 1.8	17.3 ± 1.2	14.8 ± 1.9	11.9 ± 1.5	11.4 ± 1.6	↑↑↑ *^e^*	16	>256	8 (32)	2
**2**	0	2.68	76.0	21.0 ± 2.1	11.5 ± 0.9	8.0 ± 0.9	9.0 ± 1.0	15.9 ± 2.1	↑↑↑ *^e^*	16	>256	8 (32)	2
**3**	0	3.13	76.0	15.3 ± 1.5	11.3 ± 1.3	6.3 ± 0.7	4.4 ± 0.7	10.1 ± 1.6	↑↑↑ *^e^*	16	>256	4 (32)	4
**4**	−2	−1.63	139.6	>50	>50	>100	>100	>50	- *^f^*	16	>256	16 (>256)	1
**5**	−1	0.75	102.3	>50	>50	>100	>100	6.1 ± 0.4	↑ *^g^*	16	>256	2 (16)	8
**6**	−1	0.88	102.3	>50	>50	>100	37.3 ± 0.6	16.1 ± 1.1	↑ *^g^*	16	>256	4 (16)	4
**7**	−1	1.32	102.3	>50	>50	>100	21.0 ± 1.3	14.7 ± 1.6	↑ *^g^*	16	>256	4 (16)	4
**8**	0 *^h^*	0.34	134.2	>50	>50	>100	>100	>50	- *^f^*	16	>256	8 (64)	2
**9**	0	5.69	94.1	>50	26.2 ± 1.5	6.4 ± 0.6	12.6 ± 1.2	7.6 ± 0.3	- *^f^*	16	>256	16 (>256)	1
**10**	0	4.74	85.3	>50	>50	>50	>100	>50	- *^f^*	16	>256	16 (>256)	1
**11**	0 *^h^*	1.34	161.9	>50	>50	>100	>100	>50	- *^f^*	16	>256	16 (>256)	1
**12**	+1	4.37	68.2	>50	>50	>100	>100	>50	- *^f^*	16	>256	8 (64)	2
**13**	+1	3.98	71.5	>50	>50	>100	>100	>50	- *^f^*	16	>256	8 (64)	2
**14**	+1	4.11	71.5	>50	>50	>100	>100	>50	- *^f^*	16	>256	8 (32)	2

*^a^* Inhibition of efflux pump activity in *P. aeruginosa*; *^b^* Minimum inhibitory concentration; *^c^* Numbers in parentheses indicate the amount of the compounds used in combination with ATM; *^d^* Fold decreases in the MIC of ATM upon combination with the compounds; *^e^* Stronger inhibition of the efflux pump activity than the positive control, CCCP; *^f^* No inhibition of efflux pump activity; *^g^* Weaker inhibition of the efflux pump activity than the positive control, CCCP; *^h^* Zwitterion. All experiments were performed in triplicate, and data are presented as mean ± standard deviation (SD).

**Table 2:** Antibiotic-potentiating activity (reduction of MIC of ATM) of compound **5** against 26 carbapenemase-producing *P. aeruginosa* (CPPA) isolates *^a^*.

Strain	MIC (mg/L) of ATM in Combination with Various Amounts of 5 (mg/L)								ATM/5 Combination with Lowest FICI *^b^*			Susceptibility to ATM	
	0	1	2	4	8	16	32	64	FICI (ATM/5) *^c^*	Activity *^d^*	FD *^e^*	ATM Alone *^f^*	ATM/5 *^g^*
PA-009, *bla*_GES_	32	32	16	16	8	8	4	4	0.28 (8/8)	S	4	R	S (8)
PA-014, *bla*_GES_	16	16	16	8	8	4	4	4	0.28 (4/16)	S	4	I	S (4)
PA-035, *bla*_GES_	8	8	4	4	2	2	2	2	0.27 (2/8)	S	4	S	S
PA-003, *bla*_VIM_	32	16	16	16	16	16	16	16	0.50 (16/1)	I	2	R	I (1)
PA-002, *bla*_IMP_	32	16	16	8	8	4	4	2	0.26 (8/4)	S	4	R	S (4)
PA-016, *bla*_IMP_	32	32	16	16	8	8	4	4	0.27 (8/8)	S	4	R	S (8)
PA-021, *bla*_IMP_	1	1	0.5	0.5	0.5	0.5	0.5	0.5	0.50 (0.5/2)	I	2	S	S
PA-027, *bla*_IMP_	32	32	16	8	4	2	2	2	0.26 (8/4)	S	4	R	S (4)
PA-029, *bla*_IMP_	32	32	32	16	16	8	8	8	0.28 (8/16)	S	4	R	S (16)
PA-030, *bla*_IMP_	64	32	32	16	16	8	8	4	0.26 (16/4)	S	4	R	S (16)
PA-032, *bla*_IMP_	4	4	4	4	2	1	1	1	0.28 (2/8)	S	4	S	S
PA-017, *bla*_NDM_	16	8	8	4	4	2	2	1	0.26 (4/4)	S	4	I	S (4)
PA-025, *bla*_NDM_	16	16	16	8	8	4	2	2	0.28 (4/16)	S	4	I	S (4)
PA-026, *bla*_NDM_	8	4	4	2	2	1	1	1	0.26 (2/4)	S	4	S	S
PA-028, *bla*_NDM_	8	4	2	2	2	1	1	1	0.25 (2/2)	S	4	S	S
PA-031, *bla*_NDM_	4	2	2	2	1	1	1	1	0.27 (1/8)	S	4	S	S
PA-033, *bla*_NDM_	2	2	2	2	1	1	1	1	0.52 (1/8)	I	2	S	S
PA-034, *bla*_NDM_	16	16	16	8	4	4	4	4	0.27 (4/8)	S	4	I	S (4)
PA-036, *bla*_NDM_	16	16	16	8	4	4	4	4	0.27 (4/8)	S	4	I	S (4)
PA-038, *bla*_NDM_	16	16	16	8	4	4	4	4	0.27 (4/8)	S	4	I	S (4)
PA-039, *bla*_NDM_	16	16	16	8	4	4	4	4	0.27 (4/8)	S	4	I	S (4)
PA-040, *bla*_NDM_	4	4	4	2	2	2	1	1	0.31 (1/32)	S	4	S	S
PA-041, *bla*_NDM_	4	4	4	2	2	2	1	1	0.31 (1/32)	S	4	S	S
PA-044, *bla*_NDM_	16	16	16	8	4	4	4	4	0.27 (4/8)	S	4	I	S (4)
PA-037, *bla*_IMP/NDM_	4	2	2	2	1	1	1	1	0.27 (1/8)	S	4	S	S
PA-042, *bla*_IMP/NDM_	2	2	2	1	1	1	1	1	0.51 (1/4)	I	2	S	S

*^a^* Cells corresponding to the bacterial strains sensitive to ATM alone or in combination with **5** are shaded; *^b^* FICI = Fractional Inhibitory Concentration Index. The FICI values were calculated for every ATM/**5** combination, and the data corresponding to the ATM/**5** combination with the lowest FICI values are shown; *^c^* Lowest FICI values. Numbers in parentheses indicate concentrations of ATM and **5** that resulted in the lowest FICI; *^d^* S (Synergy, FICI ≤ 0.5), I (indifference, 0.5 < FICI ≤ 4), A (antagonism, FICI > 4); *^e^* Fold decreases in the MICs of ATM upon combination with **5**. *^f^* S (sensitive, MIC ≤ 8 mg/L), I (intermediate, MIC = 16 mg/L), R (resistant, MIC ≥ 32 mg/L); *^g^* Numbers in parentheses indicate minimum concentrations of **5** (mg/L) to make *P. aeruginosa* sensitive to ATM.

**Table 3:** MIC_50_ and MIC_90_ values of ATM at increasing concentrations of **5** *^a^*.

Conc. of 5 (mg/L)	All CPPA *^b^* (n = 26)		ATM-Resistant CPPA (n = 15)		ATM-Susceptible CPPA (n = 11)	
	MIC_50_	MIC_90_	MIC_50_	MIC_90_	MIC_50_	MIC_90_
0	16	32	16	64	4	8
1	16	32	16	32	4	8
2	16	32	16	32	2	4
4	8	16	8	16	2	4
8	4	16	8	16	2	2
16	4	8	4	16	1	2
32	2	8	4	16	1	2
64	2	8	4	16	1	2

*^a^* MIC_50_ and MIC_90_ were defined as concentrations of ATM that inhibited 50% and 90% of the isolates from each pathogen group, respectively; *^b^* CPPA, carbapenemases-producing *P. aeruginosa.*

**Table 4:** Comparison of the antibacterial efficacy of ATM-**5** with other β-lactam/β-lactamase inhibitors against 26 carbapenemase-producing *P. aeruginosa* Isolates *^a^*.

	ATM-5	Ceftazidime-Avibactam	Ceftolozane-Tazobactam	Imipenem -Cilastatin -Relebactam
MIC_50_, mg/L	4	>256	256	128
MIC_90_, mg/L	16	>256	>256	>256
Range, mg/L	0.5–16	4–>256	1–>256	0.25–>256
Susceptibility, % (n/N)	88 (23/26)	14 (4/26)	10 (2/26)	14 (4/26)

*^a^* Concentrations of β-lactamase inhibitors were 4 mg/L for avibactam, tazobactam, and relebactam, and 8 mg/L for compound **5**. The susceptibility breakpoints were 8 mg/L for ceftazidime-avibactam and ATM-**5**, 4 mg/L for ceftolozane-tazobactam, and 2 mg/L for imipenem-relebactam, respectively.

**Table 5:** Reduction of the mean bacterial loads after treatment of ATM alone or in combination with 8 mg/L of **5**.

Bacteria	ATM Conc.	Synergistic Activity *^a^*				Bactericidal Activity *^b^*		
		Log CFU/mL				ATM + 5 (Log CFU/mL)		
		ATM Only	ATM + 5 *^c^*	Diff. *^d^*	Interpretation	Initial Inoculum	Diff. *^e^*	Interpretation
Wild type	1/4 MIC	9.57	5.80	−3.77	Synergy	5.84	−0.04	Bacteriostatic
	1/2 MIC	9.31	5.80	−3.51	Synergy	5.84	−0.04	Bacteriostatic
	1 MIC	8.02	0.27	−7.75	Synergy	5.84	−5.57	Bactericidal
	2 MIC	0.17	0.00	−0.17	–	5.84	−5.84	Bactericidal
PA-002, *bla*_IMP_	1/4 MIC	9.48	7.71	−1.77	–	5.45	2.26	–
	1/2 MIC	9.02	6.66	−2.36	Synergy	5.45	1.21	–
	1 MIC	8.15	3.03	−5.12	Synergy	5.45	−2.42	Bacteriostatic
	2 MIC	5.50	0.00	−5.5	Synergy	5.45	−5.45	Bactericidal
PA-003, *bla*_VIM_	1/4 MIC	10.18	10.08	−0.1	–	5.73	4.35	–
	1/2 MIC	9.88	9.84	−0.04	–	5.73	4.11	–
	1 MIC	9.13	0.23	−8.9	Synergy	5.73	−5.5	Bactericidal
	2 MIC	6.40	0.00	−6.4	Synergy	5.73	−5.73	Bactericidal
PA-014, *bla*_GES_	1/4 MIC	9.33	8.83	−0.5	–	4.95	3.88	–
	1/2 MIC	8.83	6.60	−2.23	Synergy	4.95	1.65	–
	1 MIC	7.68	2.63	−5.05	Synergy	4.95	−2.32	Bacteriostatic
	2 MIC	2.45	0.15	−2.3	Synergy	4.95	−4.8	Bactericidal
PA-017, *bla*_NDM_	1/4 MIC	10.74	9.97	−0.77	–	5.00	4.97	–
	1/2 MIC	9.97	6.52	−3.45	Synergy	5.00	1.52	–
	1 MIC	6.52	0.25	−6.27	Synergy	5.00	−4.75	Bactericidal
	2 MIC	0.12	0.00	−0.12	–	5.00	−5.00	Bactericidal

*^a^* Synergy is defined as a ≥ 2 log_10_ decrease in CFU/mL for the combination of ATM and **5** compared to ATM alone after 24 h of incubation. *^b^* Bactericidal and bacteriostatic activities of ATM/**5** combination were defined as a ≥ 3 log_10_ reduction and <3 log_10_ reduction in CFU/mL, respectively, after 24 h of incubation compared to the initial inoculum. *^c^* Concentration of **5** was fixed at 8 mg/L. *^d^* Differences in the log CFU/mL of the bacteria treated with ATM + **5** and ATM only. *^e^* Difference in the log CFU/mL of the bacteria treated with ATM + **5** before and after 24 h of incubation.

**Table 6:** Descriptive statistics of the observed pharmacokinetic parameters of **5** *^a^*.

Parameters	Treatment	
	Intravenous (5 mg/kg)	Oral (10 mg/kg)
t_1/2_ (h)	0.92 ± 0.42	1.28 ± 0.53
t_max_ (h)	NA	2.00
C_max_ (mg/L)	7.51 ± 1.34	0.29 ± 0.14
AUC_0–last_ (mg·h/L)	3.60 ± 0.59	1.25 ± 0.57
AUC_0–∞_ (mg·h/L)	3.61 ± 0.59	1.28 ± 0.54
MRT_0–last_ (h)	0.45 ± 0.02	2.87 ± 0.25
MRT_0–∞_ (h)	0.46 ± 0.02	3.13 ± 0.35
C_0_ (mg/L)	11.14 ± 2.39	NA
V_ss_ (L/kg)	0.65 ± 0.09	NA
V_z_ (L/kg)	1.84 ± 0.76	NA
Cl (mL/min/kg)	23.55 ± 4.12	NA
F (%)	NA	17.30 ± 7.88

*^a^* Abbreviations: AUC_0–last_, area under the curve (AUC) up to the last quantifiable time-point; AUC_0–∞_; AUC from time of dose extrapolated to infinity; Cl, clearance; C_0_; back-extrapolated plasma drug concentration at time zero following bolus intravenous injection; C_max_, maximal plasma concentration; F, fraction absorbed (bioavailability); MRT_0–last_, from time of dose to the last concentration time point; MRT_0–∞_, from time of dose extrapolated to infinity; t_1/2_, half-life; t_max_, time from dose to C_max_; NA, not applicable. V_ss_, volume in steady state; V_z_, volume in terminal state.

## Data Availability

Data are contained within the article.

## Supplementary Materials

The following supporting information can be downloaded at: https://www.mdpi.com/article/10.3390/antibiotics13121202/s1, Table S1: BL/BLi combinations in use or under development; Table S2: MICs and antimicrobial susceptibilities of various antimicrobial agents against 26 carbapenemase-producing *P. aeruginosa* isolates; Table S3: Cumulative percentage of inhibition of all CPPA isolates (n = 26) and 15 ATM-resistant CPPA isolates by ATM for increasing concentrations of **5**; Figure S1: Cumulative percentage of inhibition of 26 CPPA isolates by ATM for increasing concentrations of **5**; Figure S2: Cumulative percentage of inhibition of 15 ATM-resistant CPPA isolates by ATM for increasing concentrations of **5**.
