# Supplementary material for: 7-O-Carboxylic Acid-Substituted 3-O-Alkyl Difluoroquercetin; An Aztreonam-Potentiating Agent Against Carbapenemase-Producing Pseudomonas aeruginosa Through Simultaneous Inhibition of Metallo-β-Lactamase and Efflux Pump
Source: Antibiotics (Basel). 2024 Dec 10;13(12):1202. doi: 10.3390/antibiotics13121202 (PMC11672637; doi:10.3390/antibiotics13121202)
Supplement: Supplementary file 1 [file antibiotics-13-01202-s001.zip › antibiotics-3316357-supplementary.pdf]

Supplementary Material

7-*O*-Carboxylic Acid-Substituted 3-*O*-Alkyl Difluoroquercetin; An Aztreonam-Potentiating Agent Against Carbapenemase-Producing *Pseudomonas aeruginosa* Through Simultaneous Inhibition of Metallo-β-Lactamase and Efflux Pump

Seongyeon Lee [1,†], Taegum Lee [1,†], Mi Kyoung Kim [1], Joong Hoon Ahn [1], Seri Jeong [2], Ki-Ho Park [3,*] and Youhoon Chong [1,*]

[1] Department of Bioscience and Biotechnology, Bio/Molecular Informatics Center, Konkuk University, Hwayang-dong, Gwangjin-gu, Seoul 143-701, Republic of Korea; tjddus98@konkuk.ac.kr (S.L.); co4121@konkuk.ac.kr (T.L.); mkkim@konkuk.ac.kr (M.K.K.); jhahn@konkuk.ac.kr (J.H.A.)

[2] Department of Laboratory Medicine, Hallym University College of Medicine, Chuncheon 24252, Republic of Korea; hehebox@naver.com

[3] Department of Infectious Disease, Kyung Hee University School of Medicine, Seoul 02447, Republic of Korea

* Correspondence: parkkiho@khu.ac.kr (K.-H.P.); chongy@konkuk.ac.kr (Y.C.); Tel.: +82-2-958-2966 (K.-H.P.); +82-2-2049-6100 (Y.C.)

† These authors contributed equally to this work.

Table of Contents

1. **Table S1.** BL/BLi combinations in use or under development..S3
2. **Table S2.** MICs and antimicrobial susceptibilities of various antimicrobial agents against 26 carbapenemase-producing *P. aeruginosa* isolates………………………..……………...S4
3. **Table S3.** Cumulative % inhibition of all CPPA isolates (n = 26) and 15 ATM-resistant CPPA isolates by ATM for increasing concentrations of **5**……………………...……..S5
4. **Figure S1.** Cumulative %-inhibition of 26 CPPA isolates by ATM for increasing concentrations of **5**. The % inhibition of growth of CRPA is presented on the y axis with the MIC for ATM on the x axis as related to the added concentration of compound **5** in differently colored lines……………………………………..………………………….S6
5. **Figure S2.** Cumulative % inhibition of 15 ATM-resistant CPPA isolates by ATM for increasing concentrations of **5**. The % inhibition of growth of ATM-resistant CRPA is presented on the y axis with the MIC for aztreonam on the x axis as related to the added concentration of compound **5** in differently colored lines………………………………...S7

Table S1. BL/BLi combinations in use or under development.[1]

BL/BLi	BLi activity (Ambler Class)				Status	Indication
	A	B	C	D		
FEP/EMT	+	–	+/–	+/–	Approved (2024)	cUTI
SUL/DUR	+	–	+	+	Approved (2023)	HABP/VAP
IPM/CLN/REL	+	–	+	+/–	Approved (2019)	cUTI, cIAI, ABP/VAP
MEM/VAB	+	+/–	+	–	Approved (2017)	cUTI
CAZ/AVI	+	–	+	+/–	Approved (2015)	cUTI, cIAI, ABP/VAP
FEP/Tanibobactam	+	+	+	+	Phase 3 (2024)	cUTI
ATM/AVI[2]					Phase 3 (2023)	cIAI, HABP/VAP
FEP/Zidebactam	+	+	+	+	Phase 3 (recruiting)	cUTI
MEM/Nacubactam<br>CAZ/Nacubactam	+	–	+	+/–	Phase 3 (recruiting)	cUTI
CTB/Xeruborbatam[3]	+	+	+	+	Phase 1 (2022)	cUTI
CPD/ETX0282[4]	+	–	+	+	Phase 1 (2021)	cUTI
CTB/ARX1796[5]	+	–	+	+	Preclinical	
CTB/VNRX-7148[6]	+	–	+	+	Preclinical	

[1] Abbreviations: FEP (cefepime); EMT (enmetazobactam); SUL (sulbactam); DUR (durlobactam); REL (relebactam); IPM (imipenem); CLN (cilastatin); MEM (meropenem); VAB (vaborbactam); CAZ (ceftazidime); AVI (avibactam); ATM (aztreonam); CTB (ceftibuten); CPD (cefpodoxime); cUTI (complicated urinary tract infection); HABP/VAP (hospital-acquired and ventilator-associated bacterial pneumonia); cIAI (complicated intra-abdominal infection); CRE (carbapenem-resistant Enterobactericeae). [2] ATM is stable to class B while AVI inhibits classes A, C, and D. [3] Broad-spectrum boronic acid BLi (QPX7728). [4] Orally available diazabicyclooctane BLi. [5] Oral prodrug of avibactam. [6] Orally available boronic acid BLi.

Table S2. MICs and antimicrobial susceptibilities of various antimicrobial agents against 26 carbapenemase-producing *P. aeruginosa* isolates[a]

Strains	Carbapenemase	MIC value (mg/L)									
		PIP/TZB	CAZ/AVI	CAZ	FEP	ATM	IPM	MEM	AMK	GEN	CIP
PA-009	GES	>64/4 (R)	8/4 (S)	>16 (R)	>16 (R)	32 (R)	>8 (R)	>32 (R)	16 (S)	8 (I)	>2 (R)
PA-014	GES	>64/4 (R)	8/4 (S)	>16 (R)	>16 (R)	16 (I)	>8 (R)	>32 (R)	8 (S)	>8 (R)	>2 (R)
PA-035	GES	64/4 (I)	4/4 (S)	>16 (R)	4 (S)	8 (S)	8 (R)	16 (R)	≤4 (S)	>8 (R)	>2 (R)
PA-003	VIM	>64/4 (R)	4/4 (S)	4 (S)	>16 (R)	16 (I)	>8 (R)	16 (R)	>32 (R)	>8 (R)	>2 (R)
PA-002	IMP	>64/4 (R)	>8/4 (R)	>16 (R)	>16 (R)	32 (R)	>8 (R)	>32 (R)	>32 (R)	>8 (R)	>2 (R)
PA-016	IMP	>64/4 (R)	>8/4 (R)	>16 (R)	>16 (R)	32 (R)	>8 (R)	>32 (R)	>32 (R)	>8 (R)	>2 (R)
PA-021	IMP	16/4 (S)	>8/4 (R)	>16 (R)	16 (R)	1 (S)	>8 (R)	>32 (R)	16 (S)	>8 (R)	>2 (R)
PA-027	IMP	64/4 (I)	>8/4 (R)	>16 (R)	>16 (R)	32 (R)	>8 (R)	>32 (R)	>32 (R)	>8 (R)	>2 (R)
PA-029	IMP	>64/4 (R)	>8/4 (R)	>16 (R)	>16 (R)	32 (R)	>8 (R)	>32 (R)	>32 (R)	>8 (R)	>2 (R)
PA-030	IMP	>64/4 (R)	>8/4 (R)	>16 (R)	>16 (R)	32 (R)	>8 (R)	>32 (R)	>32 (R)	>8 (R)	>2 (R)
PA-032	IMP	>64/4 (R)	2/4 (S) (R)	8 (S)	16 (R)	4 (S)	>8 (R)	32 (R)	≤4 (S)	≤2 (S)	0.25 (S)
PA-017	NDM	>64/4 (R)	>8/4 (R)	>16 (R)	>16 (R)	16 (I)	>8 (R)	>32 (R)	>32 (R)	>8 (R)	>2 (R)
PA-025	NDM	>64/4 (R)	>8/4 (R)	>16 (R)	>16 (R)	16 (I)	>8 (R)	>32 (R)	>32 (R)	>8 (R)	>2 (R)
PA-026	NDM	8/4 (S)	2/4 (R)	>16 (R)	8 (S)	8 (S)	4 (I)	≤0.25 (S)	≤4 (S)	≤2 (S)	≤0.13 (S)
PA-028	NDM	>64/4 (R)	>8/4 (R)	>16 (R)	>16 (R)	4 (S)	>8 (R)	>32 (R)	>32 (R)	>8 (R)	>2 (R)
PA-031	NDM	>64/4 (R)	>8/4 (R)	>16 (R)	>16 (R)	8 (S)	>8 (R)	>32 (R)	>32 (R)	>8 (R)	>2 (R)
PA-033	NDM	>64/4 (R)	>8/4 (R)	>16 (R)	>16 (R)	2 (S)	>8 (R)	>32 (R)	>32 (R)	>8 (R)	>2 (R)
PA-034	NDM	>64/4 (R)	>8/4 (R)	>16 (R)	>16 (R)	16 (I)	>8 (R)	>32 (R)	>32 (R)	>8 (R)	>2 (R)
PA-036	NDM	>64/4 (R)	>8/4 (R)	>16 (R)	>16 (R)	16 (I)	>8 (R)	>32 (R)	>32 (R)	>8 (R)	>2 (R)
PA-038	NDM	>64/4 (R)	>8/4 (R)	>16 (R)	>16 (R)	16 (I)	>8 (R)	>32 (R)	>32 (R)	>8 (R)	>2 (R)
PA-039	NDM	>64/4 (R)	>8/4 (R)	>16 (R)	>16 (R)	64 (R)	>8 (R)	>32 (R)	≤4 (S)	≤2 (S)	>2 (R)
PA-040	NDM	>64/4 (R)	>8/4 (R)	>16 (R)	>16 (R)	4 (S)	>8 (R)	>32 (R)	>32 (R)	>8 (R)	>2 (R)
PA-041	NDM	>64/4 (R)	>8/4 (R)	>16 (R)	>16 (R)	4 (S)	>8 (R)	>32 (R)	>32 (R)	≤2 (S)	>2 (R)
PA-044	NDM	>64/4 (R)	>8/4 (R)	>16 (R)	>16 (R)	16 (I)	>8 (R)	>32 (R)	>32 (R)	>8 (R)	>2 (R)
PA-037	IMP, NDM	>64/4 (R)	>8/4 (R)	>16 (R)	>16 (R)	2 (S)	>8 (R)	>32 (R)	>32 (R)	>8 (R)	>2 (R)
PA-042	IMP, NDM	>64/4 (R)	>8/4 (R)	>16 (R)	>16 (R)	2 (S)	>8 (R)	>32 (R)	>32 (R)	>8 (R)	>2 (R)

[a] S, I, and R indicates susceptible, intermediate, and resistant, respectively.

Abbreviations: AMK, amikacin; ATM, aztreonam; CAZ-AVI, ceftazidime-avibactam; CIP, ciprofloxacin; FEP, cefepime; GEN, gentamicin; IPM, imipenem; MEM, meropenem; PIP/TZB, piperacillin-tazobactam.

Table S3. Cumulative % inhibition of all CPPA isolates (n = 26) and 15 ATM-resistant CPPA isolates by ATM for increasing concentrations of **5**.

**Conc. of 5 (mg/L)**	**ATM conc. (mg/L)**															
	**All CPPA (n = 26)**								**ATM-resistant CPPA (n = 15)**							
	**0.5**	**1**	**2**	**4**	**8**	**16**	**32**	**64**	**0.5**	**1**	**2**	**4**	**8**	**16**	**32**	**64**
0	0	4	12	31	38	73	96	100	0	0	0	0	0	53	93	100
1	0	4	19	35	46	81	100	100	0	0	0	0	7	67	100	100
2	4	4	23	38	46	92	100	100	0	0	0	0	7	87	100	100
4	4	8	31	46	81	100	100	100	0	0	0	7	67	100	100	100
8	4	19	38	69	88	100	100	100	0	0	0	47	80	100	100	100
16	4	27	50	81	96	100	100	100	0	0	13	67	93	100	100	100
32	4	35	54	88	96	100	100	100	0	0	20	80	93	100	100	100
64	4	38	58	92	96	100	100	100	0	7	27	87	93	100	100	100

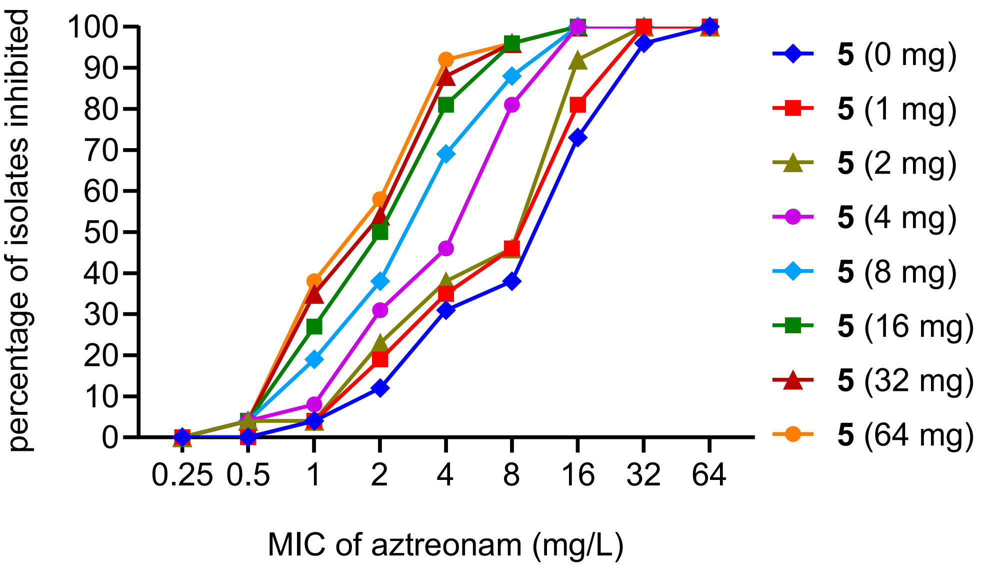

Figure S1. Cumulative %-inhibition of 26 CPPA isolates by ATM for increasing concentrations of **5**. The % inhibition of growth of CRPA is presented on the y axis with the MIC for ATM on the x axis as related to the added concentration of compound **5** in differently colored lines.

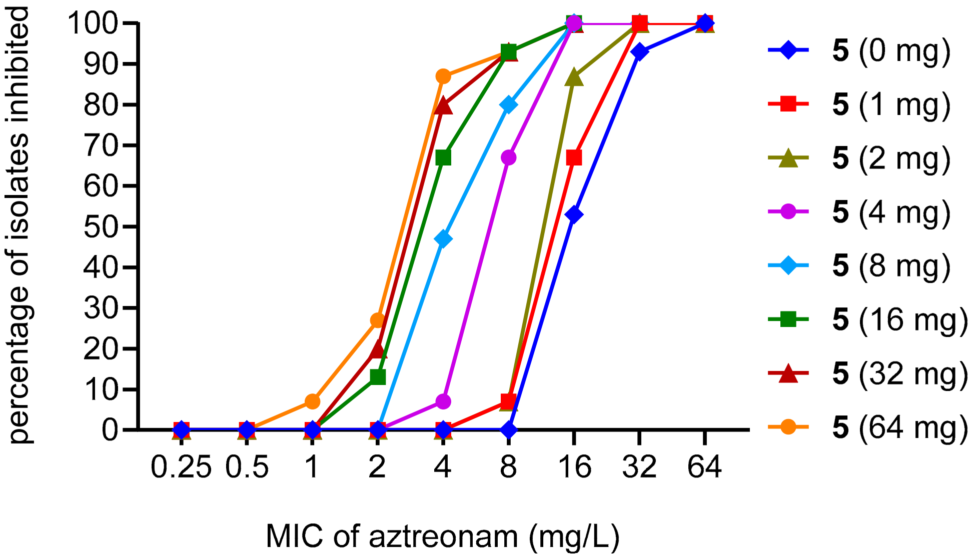

Figure S2. Cumulative % inhibition of 15 ATM-resistant CPPA isolates by ATM for increasing concentrations of **5**. The % inhibition of growth of ATM-resistant CRPA is presented on the y axis with the MIC for aztreonam on the x axis as related to the added concentration of compound **5** in differently colored lines.
